# A psychiatric disease-related circular RNA controls synaptic gene expression and cognition

**DOI:** 10.1038/s41380-020-0653-4

**Published:** 2020-01-27

**Authors:** Amber J. Zimmerman, Alexander K. Hafez, Stephen K. Amoah, Brian A. Rodriguez, Michela Dell’Orco, Evelyn Lozano, Brigham J. Hartley, Begüm Alural, Jasmin Lalonde, Praveen Chander, Maree J. Webster, Roy H. Perlis, Kristen J. Brennand, Stephen J. Haggarty, Jason Weick, Nora Perrone-Bizzozero, Jonathan L. Brigman, Nikolaos Mellios

**Affiliations:** 1 Department of Neurosciences, University of New Mexico School of Medicine, Albuquerque, NM USA; 2 Autophagy inflammation and metabolism (AIM) center, Albuquerque, NM USA; 3 Department of Psychiatry, Icahn School of Medicine at Mount Sinai, New York, NY USA; 4 Departments of Neurology and Psychiatry, Center for Genomic Medicine, Chemical Neurobiology Laboratory, Massachusetts General Hospital and Harvard Medical School, Boston, MA USA; 5 Laboratory of Brain Research, Stanley Medical Research Institute, Chevy Chase, MD USA; 6 Department of Psychiatry, Harvard Medical School, Boston, MA USA; 7 Center for Experimental Drugs and Diagnostics, Center for Genomic Medicine, Massachusetts General Hospital, Boston, MA USA; 8 Present Address: Department of Molecular and Cellular Biology, University of Guelph, Guelph, ON Canada

**Keywords:** Neuroscience, Schizophrenia, Bipolar disorder

## Abstract

Although circular RNAs (circRNAs) are enriched in the mammalian brain, very little is known about their potential involvement in brain function and psychiatric disease. Here, we show that *circHomer1a*, a neuronal-enriched circRNA abundantly expressed in the frontal cortex, derived from Homer protein homolog 1 (*HOMER1*), is significantly reduced in both the prefrontal cortex (PFC) and induced pluripotent stem cell-derived neuronal cultures from patients with schizophrenia (SCZ) and bipolar disorder (BD). Moreover, alterations in *circHomer1a* were positively associated with the age of onset of SCZ in both the dorsolateral prefrontal cortex (DLPFC) and orbitofrontal cortex (OFC). No correlations between the age of onset of SCZ and linear *HOMER1* mRNA were observed, whose expression was mostly unaltered in BD and SCZ postmortem brain. Using in vivo circRNA-specific knockdown of *circHomer1a* in mouse PFC, we show that it modulates the expression of numerous alternative mRNA transcripts from genes involved in synaptic plasticity and psychiatric disease. Intriguingly, in vivo *circHomer1a* knockdown in mouse OFC resulted in specific deficits in OFC-mediated cognitive flexibility. Lastly, we demonstrate that the neuronal RNA-binding protein HuD binds to *circHomer1a* and can influence its synaptic expression in the frontal cortex. Collectively, our data uncover a novel psychiatric disease-associated circRNA that regulates synaptic gene expression and cognitive flexibility.

## Introduction

Bipolar disorder (BD) and schizophrenia (SCZ) are multifactorial and heterogeneous psychiatric disorders with an average age of onset during late adolescence to young adulthood that together affect more than 3.5% of the US population and result in significant socioeconomic burdens [1–3]. While many studies have uncovered critical protein-coding genes associated with psychiatric disorders, such as those linked to synaptic plasticity and glutamate neurotransmission [4–8], the pathogenesis and pathophysiology of SCZ and BD remain elusive. Thus, novel molecular targets and mechanisms need to be discovered to produce clinically viable treatments. Both large and small noncoding RNAs (ncRNAs) have been recently shown to have important regulatory functions with significant implications for brain development, plasticity, and psychiatric disease [9–12]. Understanding the function of ncRNAs has led to the recognition of their ability to orchestrate the activity of complex regulatory pathways, which allows them to link multiple genetic risk factors for polygenic human disorders, such as BD and SCZ, into functional molecular networks.

Circular RNAs (circRNAs) are a novel category of long ncRNAs that are derived from the circularization and covalent joining of backspliced exons and/or introns [13–26]. CircRNAs are particularly enriched in the mammalian brain, yet, with few exceptions, lack the capacity of being translated into protein [13–26]. The recent application of improved annotation tools following deep sequencing has revealed the existence of tens of thousands of circRNAs in multiple species [16, 18, 19, 22, 23]. Some circRNAs are known to sequester microRNAs (miRNAs) by containing partial complementary sequences [17, 22, 26] and others to associate with RNA-binding proteins (RBPs) and transcription factors [15, 20]. However, the mechanism of action of the overwhelming majority of brain expressed circRNAs remains elusive. Moreover, their significance for psychiatric disorders has not yet been explored, despite the findings that brain-enriched circRNAs are being preferentially derived from genes involved in synaptic plasticity [24, 25] and the fact that circRNAs are the most resistant to degradation of the RNA species and, thus, ideal for postmortem studies. A pivotal study using mice with whole body deletions of the highly expressed circRNA CDR1as, together with its lowly expressed linear isoform, revealed changes in miRNA and activity-dependent gene expression, as well as electrophysiological and behavioral abnormalities [26], describing for the first time the potential importance of circRNAs for brain function. Moreover, recent studies utilized novel circRNA annotation approaches to extract circRNA expression data from RNA-sequencing experiments in the dorsolateral prefrontal cortex (DLPFC) of subjects with SCZ [27, 28]. Furthermore, another study uncovered alterations in a circRNA altered in the blood of patients with monopolar depression that could regulate microglial activation and depressive-like behavior in mice, thus highlighting the role of circRNAs in glial function [29]. However, nothing is known thus far about the importance of neuronal-enriched circRNAs with direct relevance to SCZ and BD in neuronal gene expression and disease-related cognition.

Previous work in induced pluripotent stem (iPS) cell-derived neuronal cultures from patients with BD has revealed the presence of hyperexcitable neuronal responses [30, 31]. Moreover, mice with a deficiency in disrupted in schizophrenia 1 (*DISC1*), a gene linked by rare variants to SCZ and mental illness [32, 33], display similar increases in neuronal excitation [34]. In addition, disturbances in the glutamatergic system and alterations in synaptic plasticity have been suggested for SCZ and BD [4–8]. HOMER protein homolog 1 (*HOMER1*) is a well-established regulator of synaptic plasticity and neuronal excitability, which has been linked to a plethora of psychiatric disorders, including SCZ and depression [35–39]. Long HOMER1 protein isoforms, such as HOMER1B and HOMER1C, dimerize and bind through their C terminus coiled-coil (CC) domains to numerous components of the postsynaptic density, such as Group1 metabotropic glutamate receptors, N-Methyl-D-aspartate (NMDA) receptor scaffolding SH3 and multiple ankyrin repeat domains proteins (SHANK), and other receptors to regulate calcium signaling in excitatory synapses [35–39]. However, the activity-dependent short isoform S (HOMER1S also known as HOMER1A) lacks the CC domain and behaves as a dominant-negative regulator of HOMER1 scaffolding capacity, thereby limiting synaptic neuronal activity and acting as a brake to neuronal excitability [35, 36, 38]. Interestingly, general *Homer1* knockout mice display symptoms reminiscent of SCZ, and specific disruption of the short or long *Homer1* isoforms results in different behavioral deficits [36, 37, 39].

Using cutting-edge circRNA methodologies in postmortem brain samples from the orbitofrontal cortex (OFC), a region of the prefrontal cortex (PFC) implicated in psychiatric disorders and responsible for high order cognitive functions, including behavioral flexibility [40–42], we have identified an abundant and activity-dependent neuronal-enriched circRNA [16, 25], *circHomer1a*, that is robustly reduced in SCZ and BD. Furthermore, we report that *circHomer1a*, which is generated from the backsplicing of four exons from the psychiatric disorder-related, synaptically expressed *HOMER1* [13, 43, 44], is also significantly downregulated in iPS cell-derived neuronal cultures from SCZ and BD patients and the DLPFC of subjects with SCZ. In addition, alterations in *circHomer1a* in both the OFC and DLPFC were found to be significantly positively associated with the age of onset of SCZ. In parallel, we show that *circHomer1a*, which is highly conserved between human and mouse, is enriched in adult mouse frontal cortex and abundantly expressed in cortical neurons but not astrocytes. Using brain region-specific in vivo knockdown of *circHomer1a* in mouse OFC we show that it is capable of regulating the expression of specific isoforms from synaptic plasticity-related genes with relevance for psychiatric disorders. Intriguingly, knockdown of *circHomer1a* in the OFC is sufficient to disrupt OFC-mediated cognitive flexibility. Moreover, we show that *circHomer1a* binds to the RBP HuD (also known as ELAV-like protein 4; ELAVL4), which results in increased synaptic *circHomer1a* expression in mouse PFC. Taken together, our study is the first to characterize the effects of a psychiatric disease-associated, neuronal-enriched circRNA on synaptic gene expression and PFC-mediated cognitive flexibility.

## Material and methods

### Animals

The Institutional Care and Use Committee (IACUC) at the University of New Mexico Health Sciences Center approved all experimental procedures. WT mice used in our study were all C57BL/6 mice (The Jackson Laboratory, Bar Harbor, ME). C57BL/6 mice overexpressing HuD under a Ca2+/calmodulin-dependent protein kinase II alpha promoter were also used (HUD-OE) [45]. HUD knockout (HuD-KO) mice were a gift from Dr Hideyuki Okano (Department of Physiology, Keio University School of Medicine, Tokyo, Japan) [46]. These mice were backcrossed with C57/BL6J for more than ten generations. Adult male mice were used for our experiments. Investigators were not blind to animal genotypes or treatment groups.

### Postmortem samples

Human postmortem brain total RNA samples from the OFC of subjects with BD (*n* = 32), SCZ (*n* = 34), and unaffected Controls (*n* = 34) were obtained from the Stanley Medical Research Institute [47]. Details on demographics are shown in Supplementary Table 1. DLPFC total RNA samples were also obtained from the same cohort.

### RNA extraction and mRNA/circRNA quantification

RNA extraction was done as shown before [48, 49]. Briefly RNA was isolated using the miRNeasy RNA isolation kit (Qiagen, Hilden, Germany). RNA quality and concentration of isolated and purchased total RNA was assayed through Nanodrop 2000 spectrophotometer and Qubit 3 (ThermoFisher Scientific). RNA was stored in a −80 °C freezer till use.

### CircRNA profiling in human postmortem brains

Profiling of circRNA expression in 100 human OFC postmortem brain (34 SCZ, 32 BD, and 34 Controls) was performed with the Arraystar Human Circular RNA Microarray (Arraystar Inc., Rockville, MD) per the manufacturer’s instructions with 13,617 probes designed to detect the unique circRNA splice junction based on numerous RNA-sequencing circRNA data [16, 19, 22, 23, 50, 51]. Briefly 800 ng of total RNA previously quantified and quality verified (see above) were treated with an aggressive RnaseR treatment (3 h at 37 °C of ribonuclease R, 20 U/μL, Epicentre, Madison WI) to digest linear RNAs and enrich for circRNA expression. The enriched for circRNAs RNA was then amplified and transcribed into fluorescent cRNA via random primers according to the Arraystar Super RNA Labelling protocol (Arraystar Inc.). The labeled circRNAs were then hybridized onto the Arraystar Human Circular RNA arrays (8 × 15 K, Arraystar, Inc.) and incubated for 17 h at 65 °C in an Agilent hybridization oven (Agilent Technologies, Santa Clara, CA). Slides were then washed and scanned with the Agilent Scanner G2505C (Agilent Technologies). Differentially altered circRNAs as shown in Supplementary Tables 2–3. All circRNA profiling data have been deposited in the Mendeley online data repository: https://data.mendeley.com/datasets/9zdhc6pmx5/1.

### Quantification of circRNA and mRNA expression

Reverse transcription was performed using the SuperScript IV First-Strand Synthesis System (ThermoFisher Scientific) with random hexamers for circRNA and oligo-dT primers for linear mRNA RNA detection. cDNA was then used together with custom made, validated, and sequence-verified circRNA and mRNA primers or TaqMan mRNA primers (ThermoFisher Scientific) for mRNA qRT-PCR. *18S rRNA* was used as a normalizer for mRNA detection, whereas *circTulp4* and *circCDR1as* were used for circRNA normalization. For mRNA qRT-PCR quantification the following formula was used: Relative value = A^Ct^*18S rRNA*^/A^Ct^mRNA^, where A = 10^(−1/primer slope). For circRNA qRT-PCR quantification the following formula was used: Relative value = A^Ct^2circRNA normalizers^(geometric mean of Ct^*circTulp4*^ and Ct^*CDR1as*^)/A^Ct^circRNA^, where A = 10^(−1/primer slope). In the cases where one circRNA normalizer (*circTulp4*) was used the formula changes to: Relative value = A^Ct^circTulp4^/A^Ct^circRNA^, where A = 10^(−1/primer slope). Lastly when no normalizers were used circRNA relative expression was calculated as: Relative value = [A^ − Ct^circRNA)^] × 10^^6^, where A = 10^(−1/primer slope). All circRNA primers were run on an agarose gel and were sequence-validated. Moreover, their resistance to RNaseR and reduced abundance in oligo-dT reverse-transcribed cDNA were tested, together with melting curve and slope analysis. Detailed information about all the primers used in our study are included in Supplementary Table 4.

Please see Supplemental Information for additional “Material and Methods”.

## Results

### Deficits in *circHomer1a* but not linear *HOMER1* mRNA expression in the OFC of both BD and SCZ and association with age of onset

**Fig. 1:**
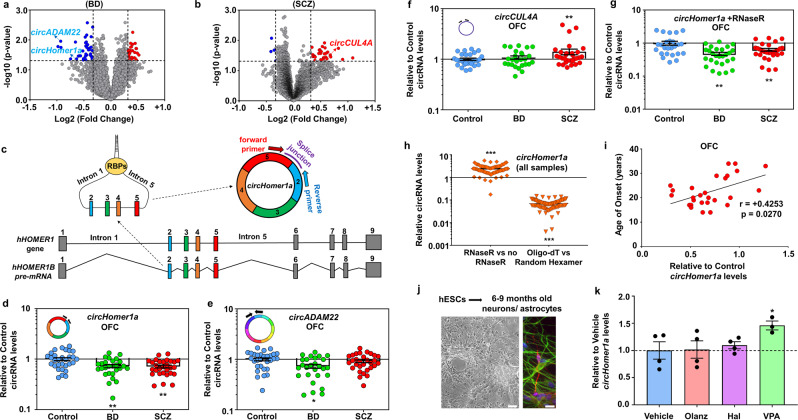
Alterations in *circHomer1a* expression in BD and SCZ OFC. Volcano plots showing differential circRNA expression in BD (**a**) and SCZ (**b**) patients vs unaffected Controls (*x*-axis = relative to control log2 fold changes; *y*-axis: negative log10 of the *p* values). Vertical lines correspond to >1.25-fold changes, and the horizontal line represents *p* < 0.05. Example of validated circRNAs is shown in the graph. **c** Schematic representation of circRNA biogenesis and detection for human *circHomer1a*. Schematic of *hHOMER1* gene and *circHomer1a* backsplicing from *HOMER1B* mRNA precursor. Complementary antisense sequences in introns 1 and 5 of *HOMER1B* are brought together by RBPs to result in the backsplicing of exons 2 and 5 to create precursor *circHomer1a* [13], which is then spliced into mature *circHomer1a*. The location for shRNAs and probes for *circHomer1a*-specific knockdown and detection, respectively, are shown. **d**–**f** Mean ± SEM relative to Control *circHomer1a* (**e**), *circADAM22* (**f**), and *circCUL4A* (**g**) levels (qRT-PCR, normalized to the geometric mean of highly expressed and unaltered *circTulp4* and *CDR1as*; see also Supplementary Fig. 3a) in the OFC of subjects with SCZ and BD. A schematic of the exonic nature of *circHomer1a* and *circADAM22* (different exons shown in different colors) and the intronic nature of *circCUL4A* is also shown. **g** Reductions in *circHomer1a* in SCZ and BD OFC via qRT-PCR in RNAseR-treated samples (no normalization, shown as relative to Control Mean ± SEM ratios). **d**–**g** **p < 0.01, based on a Univariate General Linear Model corrected for RIN, PMI, RI, and brain pH. **h** RNaseR increases the relative abundance of *circHomer1a*, whereas poly-A selection depletes *circHomer1a* expression (Mean ± SEM, ***p < 0.001, two-tailed one sample *t*-test). **i** Relative to Control changes in *circHomer1a* in the OFC of patients with SCZ are positively correlated to the age of onset of the disease. Spearman correlation coefficient and two-tailed *p* values are shown in the graph. In all postmortem data graphs, individual SCZ (red circles), BD (green circles), and Control (blue circles) sample values are shown. **j** Representative bright-field image from 6 to 9 months differentiated human embryonic stem cell (hESC)-derived very mature mixed neuronal and glial cultures. Immunofluorescence showing the presence of neurons (MAP2, red) and astrocytes (GFAP, green) is shown in the left. Scale bar = 100µm. **k** Relative to Vehicle Mean ± SEM *circHomer1a* expression (based on circRNA qRT-PCR, without normalization) following treatment with olanzapine (Olanz), Haloperidol (Hal), or valproic acid (VPA) for 2 days in human 6–9 months differentiated stem cell-derived mixed neuronal and astrocyte cultures. *p < 0.05, two-tailed one sample *t*-test. In all bar graphs the individual replicates are shown within the graph.

We employed a circRNA microarray platform that uses 13,617 circRNA splice junction probes designed based on previous RNA sequencing and circRNA annotation data [16, 19, 22, 23, 50, 51] to screen for circRNA expression in OFC RNA samples from 34 SCZ, 32 BD, and 34 unaffected control subjects from the Stanley Medical Research Institute [47] (Supplementary Table 1). We detected more than 10,000 circRNAs in these 100 RNA samples (Supplementary Fig. 1), which were all first treated with RNaseR for preferential digestion of linear transcripts (see also Supplementary “Material and Methods”). Analysis of circRNA changes in BD uncovered a subset of differentially expressed circRNAs (Fig. 1a and Supplementary Table 2) stemming from genes related to synaptic transmission, neuronal development and migration, and short-term memory (Supplementary Fig. 2a and Supplementary Table 2). On the other hand circRNAs altered in SCZ (Fig. 1b and Supplementary Table 3) were associated among others with the mitogen-activated protein kinase (MAPK/ERK) and protein kinase B (PKB/AKT) pathways (Supplementary Fig. 2b and Supplementary Table 3). Although harsh RNAseR treatment is beneficial in significantly enriching relative circRNA abundance within RNA samples by efficiently digesting all linear RNAs, it can also partially digest some circRNAs, thus making circRNA screening semiquantitative. In order to accurately validate circRNA expression we designed, validated, and sequence-verified (see circRNA splice junction sequence example in Fig. 1c, Supplementary Fig. 2c also Supplementary “Material and Methods”) circRNA-specific qRT-PCR primers aimed at the unique circRNA-specific splice junction to measure a subset of dysregulated circRNAs in BD and/or SCZ in non-RNaseR-treated samples. For normalization, we utilized the geometric mean of two highly expressed circRNAs that are unchanged in BD and SCZ based on both the circRNA array and qRT-PCR (*circTulp4*, and *CDR1as*, Supplementary Fig. 3a and Supplementary Tables 2 and 3). Our results, which were corrected for various postmortem brain demographics such as RNA integrity number (RIN), postmortem interval (PMI), brain pH, and refrigeration interval (RI) using a general linear model (see also Supplementary “Material and Methods”), revealed robust reductions in *circHomer1a*, an exonic circRNA generated from *HOMER1*, in BD as shown in the array, but also in SCZ (Fig. 1d). In addition, we validated a downregulation of *circADAM22*, a circRNA derived from the epilepsy-related gene ADAM metallopeptidase domain 22 (*ADAM22*) (Fig. 1e), only in BD. We also found that *circCUL4A*, an intronic circRNA from ubiquitin ligase Cullin-4A (*CUL4A*), was unchanged in BD but significantly increased in SCZ (Fig. 1f), similar to what was shown in the circRNA array. Of note, repeating *circHomer1a* qRT-PCR validation in RNaseR-treated samples without any normalization still showed robust reductions in both BD and SCZ, with the majority of cases exhibiting notable deficits in *circHomer1a* (Fig. 1g).

We decided to further focus on *circHomer1a*, given its notable changes in both SCZ and BD, its bona-fide circRNA identity (i.e., resistance to RNaseR treatment and depletion following poly-A tail selection—Fig. 1g, h), and its validated noncoding nature [16]. In order to determine whether postmortem demographics could influence *circHomer1a* levels in the OFC, we examined associations between changes in *circHomer1a* in both SCZ and BD and 18 separate demographics, including age, sex, and lifetime antipsychotic treatment (Supplementary Table 5). We observed no interactions with the exception of a negative correlation with duration of illness (Supplementary Table 5). On the other hand, looking at just SCZ, we did observe a significant positive correlation between changes in *circHomer1a* in the OFC and the age of onset of SCZ (Fig. 1i). To further determine the influence of antipsychotics on human neuronal and glial *circHomer1a* expression we generated very mature human pluripotent stem cell-derived mixed neuronal and glial cultures (differentiated for 6–9 months—Fig. 1j). Due to the reported accelerated neuronal maturation in stem cell-derived neuronal cultures, such a very late stage culture is expected to be of a developmental stage equivalent to postnatal human brain development and is thus more informative [52, 53]. Treatment of these cultures with either olanzapine, haloperidol, or valproic acid (VPA) for 2 days resulted in no major changes in *circHomer1a*, but a modest increase due to VPA (Fig. 1k).

CircRNA databases predict that *circHomer1a* is generated from exons 2 to 5 of *HOMER1* following backsplicing and covalent joining between exon 5 and exon 2 of the longest *HOMER1B* isoform (Fig. 1c) [43, 44]. Moreover, a previous study has suggested that *circHomer1a* backsplicing is facilitated by trinucleotide repeat-containing 6A (an RBP also known as GW182) in antisense repeat sequences in introns 1 and 5 close to the splice junction (Fig. 1c) [13]. To determine the relative *HOMER1* mRNA changes in BD and SCZ, we measured the expression of total *HOMER1* mRNA in linear RNA-enriched cDNA samples following oligo-dT reverse transcription with specific primers designed to avoid circRNA detection, and after normalization to *18S rRNA* (a reliable normalizer unchanged in BD and SCZ; Supplementary Fig. 3b, c) [10, 54, 55]. As in the case of circRNA qRT-PCR, all linear RNA data were corrected for RIN, PMI, brain pH, and RI (see also Supplementary “Material and Methods”). We found a modest reduction in total *HOMER1* expression only in *SCZ*, but no changes in BD within the OFC (Supplementary Fig. 3c). To test whether *circHomer1a* levels could be associated with linear *HOMER1* mRNA expression, we plotted *circHomer1a* changes vs total *HOMER1* mRNA. We only observed a weak positive correlation between *circHomer1a* and *HOMER1* mRNA (Supplementary Fig. 3d). Lastly, no association was found between changes in *HOMER1* mRNA in SCZ OFC and the age of onset of the disease (*r* = +0.0908, *p* = 0.6374, based on Spearman’s correlation). We thus conclude that *circHomer1a*, but not linear *HOMER1* mRNA, is notably reduced in the OFC of both BD and SCZ patients and associated with the age of onset of SCZ.

### Downregulation of *circHomer1a* but not linear *HOMER1* mRNA in SCZ DLPFC and in both SCZ and BD patient-derived neuronal cultures

**Fig. 2:**
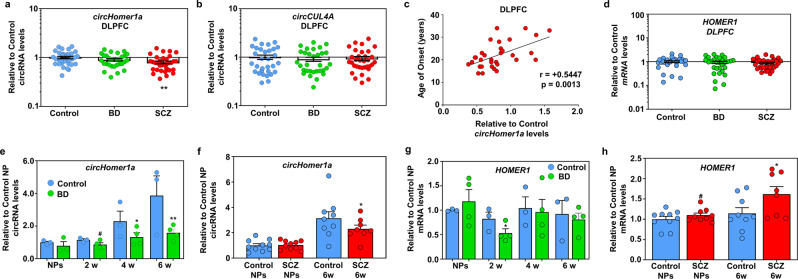
Alterations in *circHomer1a* but not linear *HOMER1* mRNA expression in SCZ DLPFC and stem cell-derived neuronal cultures of subjects with SCZ and BD. Mean ± SEM relative to Control *circHomer1a* (**a**) and *circCUL4A* (**b**) levels (qRT-PCR, normalized to the geometric mean of highly expressed and unaltered *circTulp4* and *CDR1as*; see also Supplementary Fig. 3a) in the DLPFC of subjects with BD and SCZ. **c** Relative to Control changes in *circHomer1a* in the DLPFC of patients with SCZ are positively correlated to the age of onset of the disease. Spearman correlation coefficient and two-tailed *p* values are shown in the graph. **d** Mean ± SEM relative to Control *HOMER1* mRNA levels (qRT-PCR, normalized to the highly expressed and unaltered *18S rRNA*; see also Supplementary Fig. 4c) in the DLPFC of subjects with BD and SCZ. **a**, **b**, **d** **p < 0.01, based on a Univariate General Linear Model corrected for RIN, PMI, RI, and brain pH. In all postmortem data graphs, individual SCZ (red circles), BD (green circles), and Control (blue circles) sample values are shown. Mean ± SEM relative to the mean of Control neuronal progenitors (NPs) *circHomer1a* (**e**) and *HOMER1* mRNA (**f**) levels in iPS cell-derived SCZ patient and Control (*n* = 10 Control and 9 SCZ subjects) NPs and 6-week differentiated neurons. Mean ± SEM relative to the mean of Control NPs *circHomer1a* (**g**), and *HOMER1* mRNA (**h**) levels in iPS cell-derived BD patient and Control (*n* = 3 Control and 4 BD) NPs and 2-, 4-, and 6-week differentiated neurons. **e**–**h** ^#^0.10 > *p* > 0.05, *p < 0.05, two-tailed one sample *t*-test relative to the Control of the same developmental time-point. In all bar graphs the individual replicates are shown within the graph.

In order to determine the brain region-specificity of *circHomer1a* alterations in psychiatric disorders we quantified *circHomer1a* expression in the DLPFC of BD, SCZ, and unaffected Controls from the same cohort from Stanley Medical Research Institute used for the OFC measurements. Using the same circRNA normalizers (circTulp4/CDR1as, not altered in DLPFC as well; see Supplementary Fig. 4a) and statistical analysis models as shown above for the OFC (General Linear model correcting for multiple postmortem demographics), we found that *circHomer1a* was significantly reduced in the DLPFC of only SCZ subjects (Fig. 2a). On the other hand, other circRNAs found to be altered in the OFC, such as *circCUL4A* and *circADAM22*, were unchanged in the DLPFC (Fig. 2b and Supplementary Fig. 4b). Interestingly, changes in *circHomer1a* in SCZ were positively associated with the age of onset of the disease in the DLPFC (Fig. 2c). Moreover, using the same linear mRNA quantification, normalization (*18S rRNA*—not changing at DLPFC as well; Supplementary Fig. 4c), and analysis methods as the ones used for the OFC, we found that linear *HOMER1* mRNA was not altered in the DLPFC (Fig. 2d). Furthermore, changes in *HOMER1* mRNA expression had no impact on the age of onset of SCZ (r = +0.1037, *p* = 0.5788, based on Spearman’s correlation), in contrast to *circHomer1a*. A weak positive correlation between *circHomer1a* and *HOMER1* mRNA was also found in the DLPFC (Supplementary Fig. 4d).

Furthermore, we measured the expression of *circHomer1a* and linear *HOMER1* mRNA in a large subset of iPS cell-derived neuronal progenitors (NPs) and 6-week differentiated neurons from early onset SCZ patients and controls (*n* = 9 patients/10 controls) [56], and in iPS cell-derived NPs and neuronal cultures from patients with BD and unaffected controls [10] that were differentiated for 2, 4, and 6 weeks (*n* = 4 patients and 3 controls for each developmental stage) (Fig. 2e–h). Our results revealed that *circHomer1a* was consistently downregulated in both BD and SCZ patient-derived neuronal cultures (Fig. 2e, f), but not NPs, but it appeared to be more robustly reduced in 6-week BD patient-derived neurons (Fig. 2e). In addition, the expression of *circHomer1a* increased threefold to fourfold as neurons differentiated to become synaptically active (6 weeks after differentiation vs NPs, Fig. 2e, f). Moreover, *HOMER1 mRNA* was significantly increased in 6-week SCZ iPS cell-derived neurons and displayed no changes in 6-week BD iPS cell-derived neurons in contrast to *circHomer1a* (Fig. 2g, h). Furthermore, no notable developmental changes were seen in the expression of *HOMER1* mRNA (Fig. 2g, h). Of note, NPs and 6-week iPS cell-derived neuronal cultures from the BD and SCZ cohorts, displayed similar GABAergic and excitatory neuronal gene expression, but had some differences in NPs and immature neuronal gene expression (Supplementary Fig. 5a–d). We conclude that *circHomer1a* is significantly downregulated in stem cell-derived neuronal samples from both patients with BD and SCZ and is also reduced in the DLPFC of patients with SCZ, where its changes are also positively associated with the age of onset of the disease.

### *CircHomer1a* is a neuronal-enriched circRNA abundantly expressed in the frontal cortex that binds to HuD

**Fig. 3:**
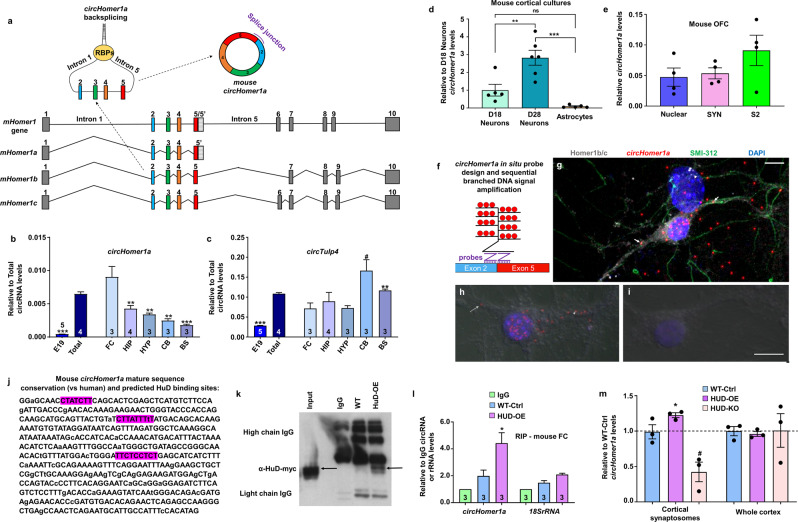
Developmental, cellular, and subcellular specificity of *circHomer1a* expression and effects of HuD binding. **a** Schematic showing mouse *Homer1* gene, *circHomer1a*, and *Homer1* pre-mRNA isoforms. Mean ± SEM mouse *circHomer1a* (**b**) and *circTulp4* (**c**) (based on qRT-PCR, without normalization) in E19 fetal (E19) and adult whole brains (Total), as well in adult FC frontal cortex, HIP hippocampus, HYP hypothalamus, CB cerebellum, and BS brainstem. **p < 0.01, ***p < 0.001, based on two-tailed one sample *t*-test compared with the mean of circRNA expression in adult whole brain. **d** Mean ± SEM relative to the mean expression in DIV18 neurons) *circHomer1a* levels (normalized to *18S rRNA*) in mouse cortical neurons of DIV18 and DIV28 of differentiation and mouse cortical astrocytes. **p < 0.01, ***p < 0.001, based on ANOVA with correction for multiple comparison. **e** Mean ± SEM relative *circHomer1a* expression (based on qRT-PCR, without normalization) in mouse OFC nuclear, crude synaptosomal (SYN), and cytoplasmic/soluble (S2) subcellular fragments. In all bar graphs, the number of replicates is shown within the graph. **f** Schematic showing *circHomer1a* in situ probe design using two probes that can bind to the exon 2/5 splice junction and detection via sequential branched DNA signal amplification. **g** Image showing the expression of *circHomer1a* in mouse neuronal cultures via in situ hybridization with two probes aiming at its splice junction (both probes need to specifically bind for a signal to be generated) and signal amplification (red). Co-Immunostaining for Homer1b/c (gray—dendritic and post synaptic) and SMI-312 (green—pan axonal) is also shown. DAPI staining is shown in blue. Scale bar = 50µm. Higher magnification from *circHomer1a* in situ hybridization (**h**) and negative control (no-probe, **i**) in mouse neuronal cultures overlaid with bright-field images. Notice the subcellular expression of *circHomer1a* in the nucleus, cytoplasm, and neurites (white arrows) of a pyramidal neuron-like cell (**h**). Scale bar = 25µm. **j** Sequence of mouse *circHomer1a* with the three predicted HuD binding sites highlighted in purple and the nucleotides that are conserved with human shown in capital. **k** Representative blot following RIP (anti-Myc) in human HuD-Myc overexpressing mouse PFC (HuD-OE-Myc) and WT littermates (WT-Ctrl). Input and IgG control is also shown. **l** Relative to IgG control *circHomer1a* levels based on qRT-PCR following RIP (anti-Myc) in human HuD-OE-Myc and WT littermates (WT-Ctrl). **m** Mean ± SEM relative to WT *circHomer1a* levels in frontal cortex synaptosomes and total frontal cortex isolates (PFC total) from HuD-OE and HUD-KO mice. **h**, **i**: *p < 0.05, **p < 0.01, two-tailed one samples *t*-test. In all bar graphs the individual replicates or the number of replicates is shown within the graph.

Despite the low evolutionary conservation of most circRNAs, the mature *circHomer1a* sequence is highly conserved between human and mouse (16, 24, 25, 43, 44; see also Supplementary Fig. 6). On the other hand, with the exception of mouse *Homer1b and Homer1a*, which have a high degree of sequence similarities to human *HOMER1B and HOMER1A,* respectively, other well-characterized mouse isoforms, such as *Homer1c*, do not appear to be much conserved (Fig. 3a and not shown, see also Fig. 1d). As far as the developmental- and brain region-specificity of mouse *circHomer1a*, we found that it was robustly upregulated from prenatal to adult total brain and it was enriched in the adult frontal cortex with lower expression in other brain regions (Fig. 3b). On the other hand, *circTulp4* was modestly upregulated in adult vs fetal brain and was similarly expressed in most brain regions with the exception of modest increases in the brainstem and cerebellum (Fig. 3c). Moreover, expression of *circHomer1a* was found to be upregulated during differentiation of mouse cortical neuronal cultures, but was barely detectable in mouse cortical astrocytic cultures (Fig. 3d). In contrast, *circTulp4*, which is known to be highly expressed in the mouse brain, was found to have the highest expression in immature neurons and moderate expression in astrocytes and mature neurons (Supplementary Fig. 7a). Subcellular fractionation and synaptosome isolation in adult mouse OFC samples revealed equal distribution of *circHomer1a* expression in synaptosomes, nuclear, and cytoplasmic/soluble fractions (Fig. 3e), which was similar to *circTulp4*, a known synapse-enriched circRNA (Supplementary Fig. 7b). Moreover, in situ hybridization for *circHomer1a* in mouse neuronal cultures using a circRNA-specific two-probe splice junction approach that also utilizes sequential branched DNA signal amplification revealed that it was enriched in pyramidal-like neurons, where it was expressed in both the nucleus, the cytoplasm, and neurites (Fig. 3f, i), which is in agreement with a previous study [25].

RBPs have been shown to bind to circRNAs and are hypothesized to contribute to circRNA intracellular trafficking. In silico analysis of RBP/circRNA interactions predicted three strong binding sites within the mature *circHomer1a* sequence for HuD (Fig. 3j), a member of the ELAV family of RBPs that has been shown to influence neurite and synaptic trafficking of neuronal mRNAs [57, 58]. Moreover, the predicted HuD binding sites were very much conserved between mouse and human (two sites had 100% sequence conservation and one had a single nucleotide change from T to C, which is not predicted to affect the binding site—Fig. 3j). Of note, we found that overall mouse mature *circHomer1a* had a 93.5% sequence conservation compared with human (Fig. 3j). To validate RBP binding to *circHomer1a,* we performed RNA immunoprecipitation (RIP) with an anti-myc antibody in the PFC of mice overexpressing a human myc-tagged HuD isoform (HuD-OE) under the control of a forebrain-specific promoter [46] (Fig. 3k). Analysis of *circHomer1a* abundance in RIP vs IgG controls revealed that HuD indeed binds to *circHomer1a* (Fig. 3k, l). Given the known role of HuD in synaptic plasticity and RNA transport [57], we decided to examine the effects of HuD overexpression and knockdown in total and synaptic *circHomer1a* levels. Our results showed that, although total *circHomer1a* expression was unchanged in HuD-OE and HuD-KO mouse frontal cortex [58], synaptic *circHomer1a* levels were significantly upregulated in the frontal cortex of HuD-OE mice and displayed a trend for reduced expression in HuD-KO mice (Fig. 3m). Given that a previous study had suggested that HuD can bind to *Homer1a* mRNA in an activity-dependent manner [58] and since multiple *Homer1* mRNA isoforms use exons 2–5 (see Fig. 3a), we quantified the capacity of *Homer1* mRNA isoforms to bind to HuD and the changes in their total and synaptic expression following OE of KO of HuD. We found that *Homer1a* binds to HuD, in accordance to the literature [58], with *Homer1b* mRNA showing a trend for enrichment following RIP and *Homer1c* shown to not bind to HuD (Supplementary Fig. 7c). Furthermore, total *Homer1a* but not *Homer1b* and *Homer1c* mRNA expression was reduced in HuD-OE mouse frontal cortex, whereas synaptic expression of all three *Homer1* isoforms was upregulated in the frontal cortex of HuD-KO mice (in contrast to *circHomer1a*, whose synaptic expression is reduced in HuD-KO mice; Supplementary Fig. 7d–f). Of note, synaptic *Homer1b* mRNA expression was found to be differentially altered in the frontal cortex of both HuD-OE and HuD-KO mice in a manner that was opposite to what was observed for *circHomer1a* (Supplementary Fig. 7e). We thus conclude that *circHomer1a* is a neuronal-enriched circRNA that is capable of binding to HuD, which can also interact with specific linear *Homer1* mRNA isoforms.

### In vivo OFC *circHomer1a* knockdown impairs OFC-mediated behavioral flexibility

**Fig. 4:**
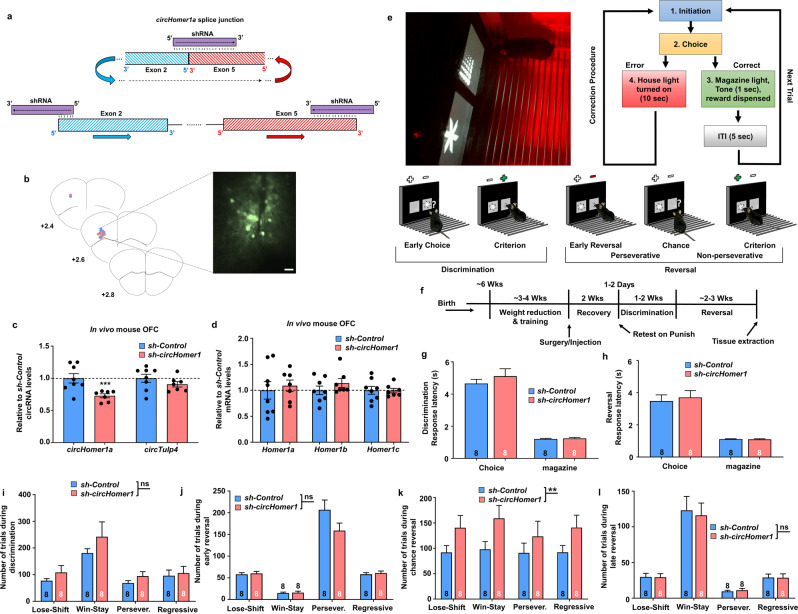
*CircHomer1a* regulates OFC-mediated reversal learning. **a** Schematic of circRNA-specific shRNA knockdown design for mouse *circHomer1a*. The shRNA targeting the *circHomer1a* splice junction is asymmetrically complimentary with the 5′ of exon 2 and the 3 of exon 5, which participate in the creation of *circHomer1a* via backsplicing and covalent joining (upper). The same shRNA does not cause have any significant complementarity with either exon 2 and exon 3 when present in any linear *Homer1* mRNA to cause degradation or miRNA-like translational inhibition and subsequent decay (only nucleotides 1–6 in the 5′ “seed sequence” of the shRNA are complementary with the 5′ of exon 2). **b** Hit-Map of injection locations for *sh-Control* (blue) and *sh-circHomer1* (pink). Coordinates are anterior from Bregma: AP + 2.6, ML ±  1.35, DV −2.7. Inset shows representative lentiviral-mediated expression of an shRNA/human synapsin promoter-driven GFP-expressing vector in mouse OFC. Scale bar = 50µm. Mean ± SEM relative to scrambled shRNA control (sh-Control) mouse *circHomer1a* (**c**) and *Homer1* mRNA isoform (**d**) levels after shRNA-mediated *circHomer1a* knockdown (sh-*circHomer1*) in mouse OFC. ^#^0.10 < *p* < 0.05, *p < 0.05, two-tailed one sample *t*-test relative to *sh-Control* mean. All data were normalized to *18S rRNA*. **e** Lower: Stages of touch-screen reversal learning behavioral paradigm. Discrimination criterion = ≥ 85% correct, early reversal = first session of reversal with performance <20%, chance reversal = 50% correct, Reversal criterion = ≥ 85% correct. Upper: Image from an example of a touch-screen-based reversal learning trial is shown on the right. Trials are initiated through a lever press [1], which leads to the onset of the pairwise stimuli on a touch sensitive screen [2]. Touch of the rewarded stimulus results in delivery of reward in the magazine [3] concomitant with 1 s tone and illumination of the magazine light. Touches at the unrewarded stimulus lead to illumination of the house light [4] with a 10 s timeout for an incorrect response. Error choices are followed by correction trials in which a subsequent initiation led to the stimuli presented in the same spatial orientation until a correct response is made to prevent side-bias and measure perseveration. **f** Behavioral paradigm intervention and injection timeline. After training, lentiviral injection with *circHomer1a* or scrambled control shRNA, and 2 weeks of recovery, discrimination, and reversal learning trials were carried out. In vivo knockdown of *circHomer1a* in mouse OFC does not alter reaction time to choose between stimuli (choice) or retrieve a reward (magazine) during both discrimination (**g**) and reversal learning (**h**). In vivo knockdown of *circHomer1a* in mouse OFC does not alter the number of trials needed for discrimination (**i**), early (**j**), and late (**l**) reversal but significantly increases the number of trials for chance reversal learning (**k**). Trial numbers are separated in lose–shift, win–stay, perseverative, and regressive. Bar graphs in **g**–**i** represent mean ± SEM and display the number of replicates within. **p < 0.01 following two-way ANOVA. In all bar graphs the individual replicates or the number of replicates are shown within the graph.

Because of the very large size of introns 1 and 5, which are predicted to be necessary for *circHomer1a* backsplicing (see also Fig. 3a) [13, 21, 43, 44], and the fact that they are also needed for linear *Homer1* expression, we are not able to specifically affect *circHomer1a* backsplicing by deleting introns 1 and 5 with genome editing. However, aiming at the unique mature *circHomer1a* splice junction between exons 5 and 2 (see Figs. 1d and 3a), we designed an shRNA that can specifically knock down the mouse mature (spliced) *circHomer1a* sequence, without directly targeting linear *Homer1* mRNAs (Fig. 4a; see also Supplementary “Material and Methods”). So as to avoid any partial inhibition on the expression of the linear mRNAs stemming from mouse *Homer1*, we used a uniquely designed circRNA-specific shRNA approach that targets the splice junction in an asymmetric way between the two exons (Fig. 4a). Such an asymmetric shRNA design does not permit any significant complementarity between the 5′ “seed” sequence of the *circHomer1a* shRNA (nucleotides 2–7) and either exon 2 or exon 5 of linear *Homer1* mRNAs, which when present could result in miRNA-like translational inhibition and subsequent mRNA decay (Fig. 4a).

To that end, we first performed in vivo *circHomer1a* knockdown in adult mouse OFC via lentiviral transduction of *circHomer1a* shRNA (*sh-circHomer1*) and scrambled control shRNA (*sh-Control*) vectors, which also expressed GFP via the human Synapsin promoter (Fig. 4b and Supplementary Fig. 8a, b). Transducing mouse neuronal cultures, we found that *sh-circHomer1* was able to reduce the expression of *circHomer1a* by approximately twofold without affecting the levels of any of the known mouse linear *Homer1* mRNA isoforms (Supplementary Fig. 8c, d). In vivo lentiviral transduction in the OFC achieved an approximate 40% reduction in *circHomer1a* levels in *sh-circHomer1* vs *sh-Control* injected mice (Fig. 4c). Given that, as described above, our shRNA approach does not interfere with backsplicing and circRNA synthesis, but acts by specifically degrading the mature exonic *circHomer1a* sequences, we did not observe, as expected from our culture data, any changes in the linear *Homer1* mRNA isoforms *Homer1a, Homer1b, and Homer1c* (Fig. 4d).

Patients with BD or SCZ exhibit cognitive deficits related to OFC function with the most notable being disturbances in cognitive flexibility, which is the capacity to promptly adapt one’s behavior when circumstances change [40–42]. Cognitive flexibility is best assessed through reversal learning tests, during which subjects are initially trained and rewarded to discriminate one visual cue from another until they reach a criterion level of performance. Then the designation of the correct (rewarded) vs incorrect visual cue gets switched and subjects are tested in their capacity to adjust their behavior [42]. Mice can be trained to perform similar OFC-dependent reversal learning behavioral tests with touch-screen learning paradigms and concomitant in vivo electrophysiological recordings [59–61]. A recent paper suggested that, during the criterion discrimination phase of a touch-screen reversal learning paradigm, OFC neuronal firing appears to track rewarded responses following a previously rewarded choice (win–stay) when behavior is well learned, but shifts to predominantly track repeated errors in early reversal and switches to track unexpected rewards at chance reversal [61]. Interestingly, NMDA receptor knockout in the OFC or lesions in the OFC can completely abrogate reversal learning [59]. Mouse *circHomer1a* is robustly increased in adult mouse brain relative to fetal brain and the frontal cortex displays the highest levels of *circHomer1a* (Fig. 3b), suggesting that deficits in *circHomer1a* in the OFC might have significant consequences. We employed a touch-screen reversal learning paradigm (Fig. 4e, f) in mice injected with *sh-circHomer1* and *sh-Control* expressing lentiviruses in the OFC. We found that *circHomer1a* knockdown in the OFC did not interfere with response latency for retrieving the reward (magazine) and time to choose stimulus (choice), suggesting no motivation and motor abnormalities, respectively (Fig. 4g, h). Interestingly, we found that *circHomer1a* knockdown significantly impaired chance reversal learning (in which the percentage of correct responding is between 30 and 60 percent—see also Fig. 4f), without influencing discrimination learning as well as the early (perseverative) and late (criterion) stages of reversal learning (Fig. 4i–l). Moreover, the increase in the number of trials needed for chance reversal in mice with in vivo *circHomer1a* knockdown were observed in all four types of choice combinations (lose–shift, win–stay, perseverative, and regressive) (Fig. 4k). Of note, there was no significant difference between *sh-circHomer1* and *sh-Control* total activity level over the entire 48 h period as shown by home-cage monitoring, indicating that *circHomer1a* knockdown does not impact locomotor activity in a familiar, home-cage environment (Supplementary Fig. 9a). Moreover, no significant differences were observed on distance traveled, duration, and average velocity of travel in the novel open field, which suggests that localized knockdown of *circHomer1a* in the OFC does not alter locomotor activity or anxiety-like behavior in a novel environment (Supplementary Fig. 9b–e). Taken together, our data suggest that modest *circHomer1a* deficits in the OFC are sufficient to impair specific stages of reversal learning but do not alter discrimination, motor functions, motivation, and anxiety-like behavior.

### In vivo knockdown of *circHomer1a* results in robust changes in alternative isoform abundance of synaptic plasticity- and psychiatric disease-associated genes

**Fig. 5:**
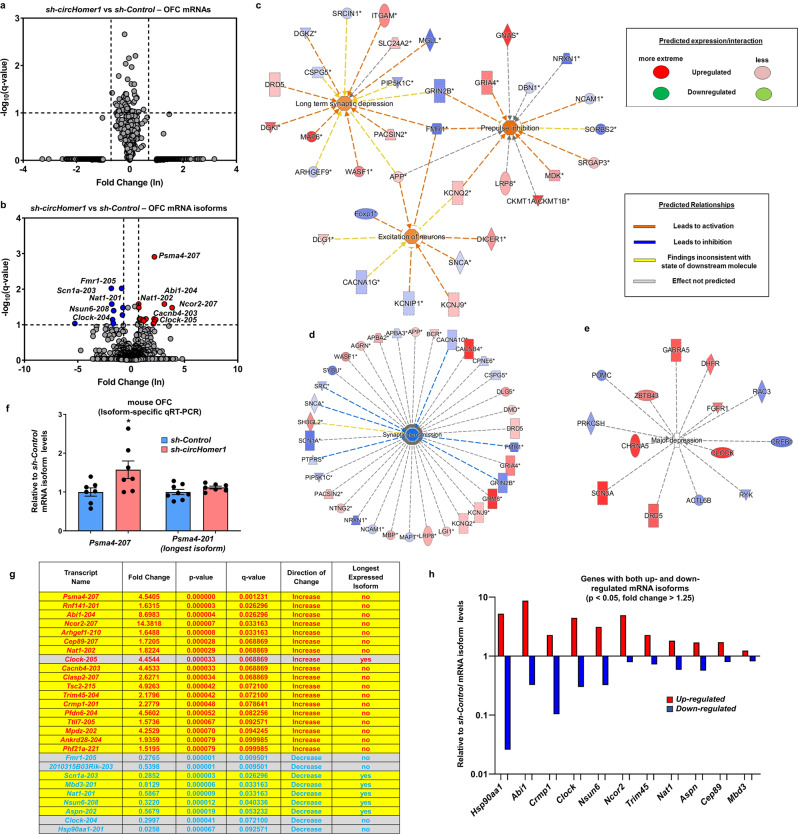
In vivo *circHomer1a* knockdown in the OFC alters the expression of mRNA isoforms from genes involved in neuronal function and psychiatric disease. Volcano plots showing differential mRNA (**a**) and mRNA isoform (**b**) expression in the OFC of *sh-circHomer1* vs *sh-Control* expressing mice (*x*-axis = relative to control log2 fold changes; y-axis: negative log10 of the *q* values). Vertical lines correspond to >2-fold changes, and the horizontal line represents *q* < 0.10. Pathway analysis of altered mRNA isoforms (**c**–**d**) or mRNAs (**e**) in *sh-circHomer1* vs *sh-Control* OFC samples (RNA sequencing) based on ingenuity pathway analysis. Information on molecular expression/interactions/relationships are shown in the graph. **f** Mean ± SEM relative to scrambled shRNA control (*sh-Control*) mouse *Psma4-207* and *Psma4-201* mRNA isoform levels after shRNA-mediated *circHomer1a* knockdown (*sh-circHomer1*) in mouse OFC. *p < 0.05, two-tailed one sample *t*-test relative to *sh-Control* mean. Data are based on isoform-specific qRT-PCR and are normalized to *18S rRNA*. **g** List of significantly altered in the OFC mRNA isoforms (*q* < 0.10, fold change > 1.25) following in vivo *circHomer1a* knockdown. Information on fold change, *p* value, *q* value, directions of change, and isoform length (yes = longest mRNA isoform in the OFC; no = not the longest expressed in the OFC isoform) is included. **h** Graph showing relative to *sh-Control* mRNA isoform levels from 11 genes that have both up- and down-regulated mRNA isoforms differentially expressed in the OF of *sh-circHomer1* mice (genes with altered mRNA isoforms from the list shown in **g** were chosen and those that displayed significant increased and decreased mRNA isoform expression include; cutoff for this comparison was dropped to *p* < 0.05).

Given that *circHomer1a* knockdown does not interfere with *Homer1* mRNA expression, we decided to perform deep RNA sequencing in OFC tissue extracted from a subset of *sh-circHomer1* and *sh-Control* expressing mice used for the reversal learning experiments described above, so as to examine any trans effects on OFC gene expression as a result of *circHomer1a* deficits. Looking at total mRNA levels in the OFC of *sh-circHomer1* and *sh-Control*, we found a very modest effect from *circHomer1a* knockdown in overall gene expression (no significant genes using a cutoff of 1.5-fold and *q* < 0.10; 19 reduced and 24 increased genes with *p* < 0.05 and 1.5-fold cutoffs—see also Fig. 5a). However, looking at alternative isoforms we found that *circHomer1a* knockdown in the OFC resulted in robust alterations (9 reduced and 18 increased genes using a cutoff of 1.5-fold and *q* < 0.10; 195 reduced and 269 increased genes with *p* < 0.05 and 1.5-fold cutoffs—see also Fig. 5b). Among these notably altered (*q* < 0.10) mRNA isoforms were isoforms from fragile X mental retardation 1 (*Fmr1*), sodium channel, voltage-gated, type I, alpha subunit (*Scn1a*), and Heat shock protein 90 kDa alpha (cytosolic), member A1 (*Hsp90aa1*) (reduced between 3- and 38-fold relative to *sh-Control*), proteasome subunit alpha type-4 (*Psma4*), Abl interactor 1 (*Abi1*), nuclear receptor co-repressor 2 (*Ncor2*), and voltage-dependent L-type calcium channel subunit beta-4 (*Cacnb4*) (increased between 4- and 14-fold relative to *sh-Control*), most of which are genes with strong links to psychiatric and neurodevelopmental disorders and synaptic function and neuronal excitability (Fig. 5b) [62–72]. Of note, two different isoforms from Circadian Locomotor Output Cycles Kaput (*Clock*), a gene involved in circadian rhythms and linked to depression and BD [73–75], were found to be differently altered (one more than threefold reduced and one more than fourfold increased) by *circHomer1a* knockdown (Fig. 5b). Importantly, analysis of potential off-target effects of *circHomer1a* knockdown via partially complementary interactions with *circHomer1a* shRNA revealed no significant results (0 mRNAs and 0 mRNA isoforms predicted to partially bind in their 3′UTR with the seed-sequence of the *circHomer1a* shRNA—*q* < 0.10 cutoff).

In order to examine the molecular pathways that could be formed following *circHomer1a* knockdown in the OFC we ran Ingenuity Pathway analysis on differentially expressed mRNA isoforms and genes (cutoff lowered to *p* < 0.05). Our results showed that differentially expressed mRNA isoforms are involved in synaptic transmission, long term synaptic depression, excitation of neurons, and prepulse inhibition (Fig. 5c, d). On the other hand, differentially expressed mRNAs were associated with major depression (Fig. 5e). Moreover, an overall significant enrichment for SCZ GWAS genes was observed in significantly altered mRNA isoforms (*p* = 0.0393, *x*^2^ = 3.095). Furthermore, using mRNA isoform-specific qRT-PCR, we validated that the *Psma4-207* mRNA isoform (but not the longest *Psma4-201* isoform) was specifically increased in the OFC following *circHomer1a* knockdown, as shown by RNA-Seq (Fig. 5f). Intriguingly, we noticed that only 1 out of 18 mRNA isoforms significantly increased in the OFC following *circHomer1a* knockdown (*q* < 0.10 and fold change >1.25) happened to be the longest expressed in the OFC isoforms per coding region sequence size (Fig. 5g). On the other hand, five out of nine of the downregulated mRNAs isoforms were the longest of the expressed in the OFC isoforms (Fig. 5g). Notably, this effect of size transcript was significant following a two-tailed chi-squared test with Yates correction (*p* = 0.0141, *x*^2^ = 6.027). Furthermore, dysregulation in 11 out of these 27 significantly altered mRNA isoforms appeared to involve two differentially altered isoforms (one increased and one decreased; cutoff reduced to *p* < 0.05 for the second isoforms) (Fig. 5h). We conclude that *circHomer1a* knockdown in the OFC results in differential expression of mRNA isoforms from genes related to synaptic function and psychiatric disease.

## Discussion

Emerging data suggest that circRNAs are enriched in the brain, expressed in synapses, and preferentially generated from synaptic-related genes. However, little is known about their importance for brain function and behavior, and more importantly for their role in brain disorders. Here, we provide novel evidence that *circHomer1a*, a highly expressed, neuronal-enriched, and evolutionary conserved circRNA originating from *HOMER1*, a gene known to regulate neuronal excitability and synaptic plasticity and linked to psychiatric disorders, is reduced in the OFC and stem cell-derived neuronal cultures of both BD and SCZ patients. Furthermore, we show that *circHomer1a* levels are also reduced in the DLPFC of subjects with SCZ and that changes in *circHomer1a* in both DLPFC and OFC are significantly positively correlated with the age of onset of SCZ. We also demonstrate that these alterations in *circHomer1a* and associations with the age of onset of SCZ are not observed in linear *HOMER1* mRNA levels, suggesting a circRNA-specific effect. Moreover, we provide evidence that *circHomer1a* is developmentally regulated and enriched in the frontal cortex, and that it binds to the RBP HuD, which in turn affects *circHomer1a* synaptic levels within the frontal cortex. Using in vivo *circHomer1a*-specific knockdown in mouse OFC we show that *circHomer1a* is necessary for OFC-mediated cognitive flexibility and that it robustly alters the expression of numerous mRNA isoforms from genes involved in synaptic function and psychiatric disease, a subset of which display differential alternative isoform alterations. Collectively, our results combine novel molecular and behavioral data to shed light into the unexplored role of circRNAs in psychiatric disease.

Given the robust reductions of *circHomer1a* in the OFC and the relevance of this brain region for cognitive flexibility, our data showing specific behavioral deficits in reversal learning following in vivo *circHomer1a* knockdown suggest the possibility that dysregulation of this circRNA in the brain of subjects with BD or SCZ could be associated with some of the cognitive disturbances observed in these psychiatric disorders. Interestingly, multiple studies suggest that alterations in reversal learning have also been observed in patients with BD and SCZ [76–80]. Moreover, the observed reductions of *circHomer1a* in both the OFC and DLPFC of patients with SCZ suggest that its alterations in SCZ could be more widespread than in BD, which is in accordance with previous reports on miRNA dysregulation in multiple PFC regions in SCZ [11]. Given the significant positive correlation between *circHomer1a* and the age of onset of SCZ in both the OFC and DLPFC (the earlier the onset the more robust the reduction in *circHomer1a*) and the alteration on numerous mRNA isoforms strongly linked to psychiatric disease and control of neuronal function, such as *Psma4*, *Fmr1*, and *Cacnb4*, it is tempting to hypothesize that deficits in *circHomer1a* within the PFC could contribute to the disturbances in synaptic plasticity and glutamatergic neurotransmission that have been observed in SCZ [4–8]. It is also possible, given the activity-dependent nature of *circHomer1a* [25], that in some cases *circHomer1a* deficits in SCZ or BD are downstream of overall disturbances in synaptic plasticity and neuronal excitability, such as those related to NMDA hypofunction, dopamine and calcium signaling, or GABAergic dysfunction. Indeed, previous studies in iPS cell-derived neuronal cultures from BD patients have suggested the existence of hyperexcitable neuronal responses, including increases in baseline excitatory postsynaptic current frequency, which could be partly ameliorated through mood stabilizer treatment [30, 31]. Future work is needed to examine upstream regulators of *circHomer1a* alterations in psychiatric disease and the potential regulatory role of *circHomer1a* in activity-dependent synaptic plasticity.

*Homer1* mRNAs are well-established effectors of neuronal excitability and synaptic plasticity, including SCZ and depression [35–39]. Our data showing that *circHomer1a*, but not linear *HOMER1* mRNA, is altered in the PFC and stem cell-derived neuronal cultures of subjects with SCZ and BD, is associated with the age of onset of SCZ, and could influence the abundance of numerous mRNA isoforms known to regulate synaptic transmission, introduce a novel potential upstream regulator of neuronal function within the PFC stemming from the *HOMER1* gene. Moreover, given that changes in neuronal activity [59, 60] have been shown to disrupt the ability of the OFC to mediate behavioral flexibility, it is tempting to hypothesize that such *circHomer1a*-mediated disturbances in OFC neuronal function-related gene expression could be implicated in the impaired reversal learning phenotype seen following in vivo *circHomer1a* knockdown in the OFC.

A common misconception is that most circRNAs act as sponges for miRNAs similar to what has been previously reported for CDR1as, which has tens of partial complementary sites for miR-7 [17, 22]. In reality, circRNA sequences are not enriched in miRNA target sites [25] and given their overall lower expression, they are unlikely to have a significant physiological impact on miRNA abundance via a single miRNA binding site. However, circRNAs are known to have many other functions ranging from effects on transcription and splicing and stability to protein expression and localization [81]. Indeed, the mature sequence of *circHomer1a* did not exhibit multiple miRNA sites for any miRNA based on in silico analysis [43]. Our findings that *circHomer1a* knockdown in the OFC preferentially upregulated the expression of shorter mRNA isoforms and resulted in differential expression of short and long isoforms for a subset of genes are suggestive of potential effects of *circHomer1a* in alternative splicing. It is tempting to hypothesize that the binding of *circHomer1a* to RBPs that regulate alternative splicing, such as HuD [82], could contribute to the observed alterations in alternative mRNA isoform expression. Future work is needed to elucidate the exact molecular mechanisms that could underlie *circHomer1a*-mediated effects in neuronal gene expression.

One limitation of our study is that it was possible to design only a single shRNA that can target the mouse *circHomer1a* splice junction in an asymmetric manner so as to not partially inhibit linear *Homer1* mRNA levels via miRNA-like partial complementarity (see also Fig. 4a). So although the shRNA used in our study was able to specifically inhibit *circHomer1a* but not linear *Homer1* mRNA expression and was not predicted to have any notable off-target effects in the OFC based on analysis of the mouse OFC RNA-sequencing data, additional methods able to specifically manipulate *circHomer1a* expression are needed to exclude the possibility of off-target effects contributing to our observed physiological and behavioral assessments. An additional limitation of our study is that we only had postmortem samples from two brain regions of a single cohort in our possession, so it was not possible to measure if the robust deficits in *circHomer1a* expression observed in the OFC could be widespread in the majority of patients with SCZ and BD. Moreover, based on our data, we do not anticipate antipsychotic treatment to directly result in notable *circHomer1a* alterations, with the exception of mood stabilizers, such as VPA, which could upregulate *circHomer1a* expression. We do consider it possible, though, that antipsychotic and mood stabilizer treatment could ameliorate some of the *circHomer1a*-mediated changes in synaptic function and neuronal excitability-related gene expression through mechanisms independent of *circHomer1a*.

Although our study is the first to examine circRNA alterations in the OFC of either BD or SCZ patients, the DLPFC of BD patients, and in stem cell-derived neuronal cultures of subjects with BD and SCZ, two recent studies used circRNA annotation approaches to pool circRNA expression data from existing RNA-sequencing databased from the DLPFC of subjects with SCZ [27, 28]. The first of these two studies using a smaller cohort found no changes in *circHomer1a* but reductions in other circRNAs and the second study that combined RNA-sequencing data from multiple cohorts found a non-significant reduction in *circHomer1a* [27, 28]. However, none of these two studies measured *circHomer1a* expression with circRNA-specific qRT-PCR, which is currently the standard method to accurate quantify circRNA expression [28, 83]. Additional studies using circRNA-specific qRT-PCR in multiple postmortem cohorts with more detailed information on antipsychotic and mood stabilizer treatment are required to determine whether alterations in *circHomer1a* are widespread in multiple brain regions in patients with psychiatric disorders and whether there are any notable effects from psychiatric drug treatment. Furthermore, in light of a recent study showing reductions in *circHomer1a* expression in postmortem brains of subjects with Alzheimer’s disease to be associated with disease-related neuropathological and cognitive dysfunction scores [84], we anticipate *circHomer1a*-mediated control of gene expression to also be of relevance for neurological disorders.

Taken together our data introduce a neuronal- and frontal cortex-enriched circRNA as a novel molecular player with links to neuropsychiatric disorders that is consistently altered in SCZ and BD OFC and patient stem cell-derived neuronal cultures and is capable of modulating synaptic gene expression and OFC-mediated cognition. Combined our work provides the first evidence supporting the importance of circRNAs altered in psychiatric disorders in disease-related disturbances in synaptic gene expression and PFC-mediated behavior.

## Supplementary information

Supplemental methods figures and tables
