# Supplementary material for: A psychiatric disease-related circular RNA controls synaptic gene expression and cognition
Source: Mol Psychiatry. 2020 Jan 27;25(11):2712–27. doi: 10.1038/s41380-020-0653-4 (PMC7577899; doi:10.1038/s41380-020-0653-4)
Supplement: Supplementary file 1 — Supplemental methods figures and tables [file 41380_2020_653_MOESM1_ESM.docx]

**SUPPLEMENTARY DATA**

**A psychiatric disease-related circular RNA controls synaptic gene expression and cognition**

Amber J. Zimmerman^1*^, Alexander K. Hafez^1*^, Stephen K. Amoah^1-2*^, Brian A. Rodriguez^1^, Michela Dell’Orco^1^, Evelyn Lozano^1^, Brigham J. Hartley^3^, Begüm Alural^4^, Jasmin Lalonde^4-5^, Praveen Chander^1^, Maree J. Webster^6^, Roy H. Perlis^7^, Kristen J. Brennand^3^, Stephen J. Haggarty^4^, Jason P. Weick^1^, Nora Perrone-Bizzozero^1^, Jonathan L. Brigman^1^, Nikolaos Mellios^1-2#^.

1) Department of Neurosciences, University of New Mexico School of Medicine, Albuquerque, NM. 2) Autophagy inflammation and metabolism (AIM) center, Albuquerque, NM, USA. 3) Department of Psychiatry, Icahn School of Medicine at Mount Sinai, New York, NY. 4) Center for Genomic Medicine, Chemical Neurobiology Laboratory, Departments of Neurology and Psychiatry, Massachusetts General Hospital and Harvard Medical School, Boston, MA, USA. 5) Current address: Department of Molecular and Cellular Biology, University of Guelph, Ontario, Canada. 6) Laboratory of Brain Research, Stanley Medical Research Institute, Chevy Chase, MD, USA. 7) Harvard Medical School, Department of Psychiatry, Boston, MA, USA; Center for Experimental Drugs and Diagnostics, Center for Genomic Medicine, Massachusetts General Hospital, Boston, MA, USA.

** Authors contributed equally.*

*#To whom correspondence should be addressed – Nikolaos Mellios, Department of Neurosciences, University of New Mexico School of Medicine, 915 Camino de Salud NE, BSMB 145, Albuquerque, NM, USA, 87131. Email: nmellios@salud.unm.edu. Tel: 505-272-8451.*

**Pages 2-9: Methods and related references.**

**Pages 9-10: Supplementary Figure legends.**

**Pages 11-19: Supplementary Figures 1-9.**

**Page 20: Supplementary Table legends.**

**Pages 21-26: Supplementary Tables S1-S4.**

**MATERIALS AND METHODS**

**Primary cultures.**

Mouse cortical astrocyte and neuronal cultures and mouse hippocampal neuronal cultures were derived from the cortices of P0-P1 C57BL/6 male and female pups (The Jackson Laboratory, Bar Harbor, ME), and were established according to previously published method (1) with minor modifications. Briefly, for mouse neuronal cultures brains were isolated and the cortex or hippocampus was dissected in ice-cold Hanks Balanced Salt Solution (HBSS) solution (Sigma-Aldrich, St. Louis, MO) supplemented with 20% FBS and NaHCO3 (4.2mM), HEPES (1mM; Sigma-Aldrich), pH 7.35, 300 mOsm. Dissected hippocampi were digested for 10 min with 0.25% Trypsin (ThermoFisher Scientific, Waltham, MA) and DNase (1500 U; Sigma-Aldrich). Tissue was washed and dissociated using fire polished Pasteur pipettes of decreasing diameter in ice cold HBSS solution. The cells were pelleted, resuspended in plating media and plated at a density of 4-5×10^5^ cells/12-mm coverslip (ThermoFisher Scientific) coated with poly-Ornithine (0.1mg/ml; Sigma-Aldrich) and laminin (5μg/ml; ThermoFisher Scientific). Cells were allowed to adhere for 20 min before addition of 0.5ml of plating media containing Neurobasal supplemented with 1X B27, 2mM Glutamax, 0.5mg/ml Pen/Strep and 5% FBS (all from ThermoFisher Scientific) for the first 24hr. Serum was eliminated from this media after 24hr and supplemented with 4µM cytosine 1-β-d-arabinofuranoside (Ara-C; Sigma-Aldrich). Neurons were then fed by replacing half the volume of spent media with fresh media without serum or Ara-C every week. Neurons on coverslips were transduced with lentivirus as previously described (1) on day 4 in culture and assayed at the days indicated in results. For primary cortical astrocytic cultures cells were pelleted, resuspended in DMEM supplemented with 10% FBS and 10% 1x PSN plated at a density of 1 x 106 cells/Tissue Culture plate (ThermoFisher Scientific). The cells were then plated in a Tissue culture plate and grown for 5-7 days until 90% confluent, then passaged using 0.25% trypsin (ThermoFisher Scientific).

**Lentiviral Transduction of *circHomer1a* shRNA in mouse neuronal cultures.**

An shRNA sequence targeting the unique exon 5/ exon 2 splice junction of mouse *circHomer1a* (GCCATTTCCACATAGGGAGCA) and a scrambled control shRNA sequence were purchased by Biosettia (San Diego, CA). The shRNA targeting mouse *circHomer1a* (TGCTCCCTATGTGGAAATGGC) was carefully designed so that it binds asymmetrically within the exon 5/ exon 2 splice junction, thus avoiding any miRNA-mediated inhibitory effects on linear *HOMER1* mRNA transcripts. Lentiviral particles (LVPs) carrying shRNAs were purchased from System Biosciences (Palo Alto, CA). LVPs were diluted from stock concentration and applied to cells on days 2-3 in culture at 1 MOI based on an estimate of 4×10^5^ cells/coverslip.

**Mouse prenatal and adult brain samples.**

Total RNA from C57BL/6 mouse whole brain of E19 fetal and adult mice and from different adult brain regions (frontal cortex, hippocampus, hypothalamus, cerebellum, brain stem) were purchased from Zyagen (San Diego, CA).

***In vivo* *circHomer1a* knockdown in mouse orbitofrontal cortex and touched-based reversal learning behavioral paradigm.**

Twenty-four male C57BL/6J mice were obtained from The Jackson Laboratory (Bar Harbor, ME). Mice were match-pair randomly assigned to either receive *circHomer1a* shRNA (sh-*circHomer1*) or a scramble shRNA Control (*sh-Control*) (n = 12 per group). Mice were housed in pairs for the extent of the experiment except for directly following surgery to allow appropriate recovery. Housing was in a temperature- and humidity-controlled vivarium under a 12 h reverse light/dark cycle (lights off at 0800 h) and they were tested during the dark (wake) phase in a red-light room. All Methods were performed in accordance with the National Institutes of Health Guide for Care and Use of Laboratory Animals and were approved by the University of New Mexico Health Sciences Center Institutional Animal Care and Use Committee.

All operant behavior was conducted in a chamber measuring 21.6 x 17.8 x 12.7 cm (model # ENV-307W, Med Associates, St. Albans, VT) housed within a sound- and light-attenuating box (Med Associates, St. Albans, VT). A solid acrylic plate was used to cover the grid floor of the chamber to facilitate ambulation. A peristaltic pump delivered 10µl of liquid strawberry milkshake (strawberry Nesquik mixed with skim milk) into a magazine. A house-light, tone generator and an ultra-sensitive lever was located on one end of the chamber, while a touch-sensitive screen (Conclusive Solutions, Sawbridgeworth, U.K.) was on the opposite side of the chamber covered by a black acrylic aperture plate, which creates two 7.5 x 7.5 cm touch areas separated by 1cm and located at a height of 0.8cm from the floor of the chamber. KLimbic Software Package v1.20.2 (Conclusive Solutions) controlled and recorded stimulus presentation and touches in the response windows. Approximately eight weeks after birth mice were food restricted and maintained at 85% free-feeding body weight and subjected to 3 days of acclimation to the behavior room and liquid reward. Mice were habituated to the operant chamber and retrieving reward from the magazine by being placed in the chamber for ≤30 minutes with liquid available in the magazine. Once a mouse retrieved at least 10 liquid reward retrievals, during a habituation session, it began the pre-training regimen. First, mice were trained to obtain reward by pressing a lever within the chamber on an FR1 schedule. Once a mouse showed willingness to press the lever and collect 30 rewards in a <30 minute-session, it was moved to touch training. During this stage, a lever press led to the presentation of a white (variously-shaped) stimulus in 1 of the 2 response windows (spatially pseudorandomized). The stimulus remained on the screen until a response was made. Touches in the blank response window had no effect, while a touch to the white stimulus window resulted in reward delivery, immediately cued by a tone and illumination of the magazine. Once a mouse was able to initiate, touch and retrieve 30 rewards in a <30 minute-session, it was moved to the final stage of pre-training. This stage was identical to touch-training except that responses at a blank window during stimulus presentation now produced a 10-second timeout, immediately signaled by illumination of the house light, to discourage indiscriminate screen responding. Errors made on this pre-training stage (as well as on discrimination and reversal, see below), were followed by correction trials in which the same stimulus and left/right position was presented until a correct response was made. Once a mouse was able to make ≥75% (excluding correction trials) of responses at a stimulus-containing window in a 30-trial session, it was moved onto discrimination testing.

After completion of training, mice were fed *ad libitum* for 2 days prior to surgery. Mice were randomly assigned to either *circHomer1a* shRNA or scramble control shRNA groups. Mice were anesthetized with isoflurane and head-fixed in a stereotaxic apparatus (1900 Stereotaxic Alignment System, David Kopf Instruments, Tujunga, CA) as previously described (2). A 33-gauge infusion cannula (Plastics One, Roanoke, VA) attached with polyurethane tubing to a Hamilton syringe (Hamilton, Reno, NV) was directed at 2 sites bilaterally targeting the orbitofrontal cortex (OFC) (AP: + 2.60, ML: ± 1.35, DV: - 2.70 to Bregma). 0.5 µL shRNA for *circHomer1* or scramble control was infused over 5 min using a pump (GenieTouch, Kent Scientific, Torrington, CT) to the depth of -2.70 and retracted to -2.60 to form a pocket. Cannula was left *in situ* for an additional 3 minutes and then retracted to -1.30 for 1 additional minute. Following the final infusion, mice were sutured, given 0.05 mL of Buprenorphine (0.03 mg/mL), and returned to their home cage with sterile bedding. Mice were given 1 week of recovery with ad libitum food and then food reduced for one more week prior to beginning discrimination testing.

Pairwise discrimination and reversal was tested as previously described (2). Mice were first trained to discriminate 2 novel, approximately equally-luminescent stimuli, presented in a spatially pseudorandomized manner, over 30-trial sessions (5-second inter-trial interval). The stimulus designated as correct was counterbalanced across mice and genotypes. Responses at the correct stimulus window resulted in a single food reward, cued by a 1-second tone and illumination of the magazine. Responses at the incorrect stimulus window resulted in a forced timeout, signaled by illumination of the house-light. Correction trials following errors were presented, with the same stimuli, in the same spatial orientation, until a correct response was made. Discrimination criterion was ≥85% correct responding out of 30 trials, excluding correction trials, over 2 consecutive sessions. Reversal training began on the session after discrimination criterion was attained. Here, the designation of correct verses incorrect stimuli was reversed for each mouse. As for discrimination, there were 30-trial daily sessions until the mice reached a criterion of ≥85% correct responding (excluding correction trials) over 2 consecutive sessions. For discrimination and reversal, the dependent variables were correction errors, reaction time (time from lever press initiation to screen touch) and magazine latency (time from screen touch to reward retrieval). In order to examine distinct phases of reversal (early perseverative, chance, and late learning), we separately analyzed errors and correction errors for sessions where performance was below 33% correct, 34-66% correct, and performance above 67% correct,, modified from previously described (3-4).

**Home cage activity and open field behavioral testing.**

After completion of the visual reversal paradigm, spontaneous home cage activity was collected in *sh-circHomer1* and *sh-Control* mice to assess behavior in a non-aversive environment. All mice were individually housed in a standard home cage with corncob bedding with ad libitum food and water and left undisturbed for a 24hr acclimation period under their normal reverse light-dark conditions (lights on at 2000h and off at 0800h). Horizontal activity was then automatically measured by photocell beam break for 48hrs using the PAS-Homecage system (San Diego Instruments, San Diego, CA). Mice were tested using a novel open field test to assess locomotor activity and anxiety-like behavior. Twenty 11-week-old male C57BL/6 mice were tested two weeks after lentiviral-mediated shRNA knockdown of *circHomer1a* or a control shRNA that was delivered to the orbitofrontal cortex as described above. For open field testing, mice were acclimated to the test room for at least 30 minutes prior to testing, and all were tested during the dark phase between 1000 and 1200h. Mice were then placed in a corner of a white Plexiglas open field chamber (29 X 29 X 29cm) (corners ~ 45 lux; center ~ 90 lux) and allowed to freely explore for 30 minutes. Distance traveled and duration in the center or the border as well as average velocity was recorded using Ethovision XT videotracking system (Noldus Information Technology, Leesburg, VA, USA).

**OFC Crude synaptosomes preparation.** Crude synaptosomes extracts were prepared as previously described (5, 6). Briefly, the tissue was immersed in 100 µL of ice cold homogenization buffer (0.32 M sucrose, 0.1 mM EDTA, 0.25 mM DTT, 2 mM HEPES, pH 7.4) supplemented with 200 U/mL RNaseOUT™ (Invitrogen) and homogenized by mechanical disruption with pestles. Nuclei and cell debris were pelleted by centrifugation for 2 min at 2000 × g. The pellet was washed a second time with homogenization buffer and the supernatants combined. Finally, the supernatant was centrifuged for an additional 10 min at 14,000 × g and the resulting pellet represented the crude synaptosome containing fraction. Total synaptosomes RNA was extracted with Trizol® (Invitrogen) according to the manufacturer's recommendations. RNA quality and quantity was determined using the Qubit (Invitrogen) spectrophotometer. To test extraction efficiency we evaluate the small brain specific RNA BC1 enrichment in the final synaptosomes extract (SYN) compared to the previous purification steps obtained during the procedure (6).

**Human pluripotent stem cell maintenance and neuronal and glial differentiation.**

hPSCs (line WA09 passage 25-35) were maintained as a monolayer in a 6 well dish in a feeder-free environment in mTeSR1 (Stem cell technologies, Vancouver, BC, Canada) on Matrigel (BD biosciences, San Jose, CA) and passaged using 1mg/ml Dispase (Sigma-Aldrich) as shown before (7). For neuronal differentiation, hPSCs from several 80-90% confluent wells were grown in suspension via a similar to the Serum Free Embryoid Body (SFEB) differentiation paradigm (8-9), so as to generate neuroepithelial cell aggregates (Neurospheres). The day of passaging cells from monolayer into suspension was defined as day 0. For neural differentiation, neurospheres were plated on day 21 onto polyornithine-laminin-coated coverslips (100μg/ml polyornithine, 10μg/ml laminin; Sigma-Aldrich) in a 24-well plate and fed every other day with Neural Differentiation Media (NDM) as shown before (65). Day 50 human pluripotent stem cell-derived neuronal cultures (hPSNs) were treated with tetrodotoxin (TTX) (1µm) and were harvested after 48 hours. For the generation of mature mixed neuronal and astrocytic cultures, cells were allowed to differentiate for 6-9 months. Mixed mature neuronal and glial cultures were then treated with either vehicle, olanzapine (100nM), haloperidol (100nM), or valproic acid (0.5mM) for 2 days (all drugs from Sigma-Aldrich).

**Induced pluripotent stem (iPS) cell reprogramming, neural progenitor cell derivation and neuronal differentiation for BD and unaffected control subjects.**

Human fibroblasts were generated from dermal skin punches obtained from three healthy control and four BD subjects enrolled in research studies in the MGH Department of Psychiatry, Center for Genomic Medicine. A non-integrative, mRNA-based technology developed by Cellular Reprogramming, Inc. (San Diego, CA) was used to reprogram de-identified, established fibroblast lines into iPS cells. iPS cells were cultured feeder-free in E8 medium (Gibco, Gaithersburg, MD) on Geltrex coated 6-well plates with ROCK inhibitor (10 µM Thiazovivin; Stemgent, Lexington, MA, USA). They were then purified using magnetic-activated cell sorting with Tra-1-60 microbeads on LS columns according vendor’s protocol (Miltenyi Biotec, Bergisch Gladbach, Germany). Neural induction was initiated when sorted cells were 10-20% confluent using the 1X Neural Induction Supplement added to Neurobasal media (Gibco). Putative neural progenitors (NPs) were further purified by PSA-NCAM, CD133 positive magnetic-activated cell sorting with negative selection against CD271 (Miltenyi Biotec). For neuronal sample collection, cells were seeded into 6-well plates at a density of 5x10^5 cells/well and differentiated for two, four, or six weeks.

**iPS cell reprogramming, neural progenitor cell derivation and neuronal differentiation for early onset SCZ and unaffected control subjects.**

Fibroblasts from skin biopsies of 9 early (childhood)-onset schizophrenia and 10 unaffected Controls were reprogrammed in Mount Sinai Icahn School of Medicine as described before (10). Briefly fibroblasts were transduced with OCT4, SOX2, KLF4, and c-MYC expressing Cytotune® Sendai viruses (ThermoFisher Scientific), then switched to human to human iPS cell media (DMEM/F12, 20% KO-Serum Replacement (v/v), 1% (v/v) GlutaMAX, 1% (v/v) nonessential amino acids (NEAA), 55 μM β‐mercaptoethanol (all ThermoFisher Scientific) and 20 ng ml−1 FGF2 (R & D Systems, 233-FB-10)) and fed daily. iPS cell colonies were then manually picked and clonally plated onto 24-well mouse embryonic fibroblast (mEF)-coated plates and maintained on mEFs in iPSC media. NPs were generated and maintained at high density, and grown within growth factor reduced Matrigel (BD Biosciences) coated plates in NP media (Dulbecco’s Modified Eagle Medium/Ham’s F12 Nutrient Mixture (ThermoFisher Scientific), 1x N2, 1x B27-RA (ThermoFisher Scientific) and 20 ng ml−1 FGF2. For inducing neuronal differentiation, media was switched to neural differentiation medium (DMEM/F12, 1xN2, 1xB27-RA, 20 ng ml−1 BDNF (Peprotech), 20 ng ml−1 GDNF (Peprotech), 1 mM dibutyryl-cyclic AMP (Sigma-Aldrich), 200 nM ascorbic acid (Sigma-Aldrich) and 1 μg ml−1 laminin (ThermoFisher Scientific) 1–2 days later. NPs were then differentiated from for 6 weeks to generate iPSC-derived neuronal cultures.

**RNA sequencing in OFC samples with *circHomer1a* knockdown**

Library preparation and RNA sequencing (RNA-seq) was carried out by Arraystar Inc. Briefly, 1ug of Total RNA was used for library construction. Libraries were constructed using Kapa Stranded RNA-seq library kit (Illumina). Paired end RNA-seq was done on an Illumina HiSeq 4000 with a read length of 150 bps. Image analysis and base calling were performed using solexa platform v1.8. Sequence quality was examined using FastQC software. Adapter trimming and filtering was performed by cut adapt software. Reads were then aligned to mouse genome GRCm28 using HiSat 2 software. Transcript abundance for each sample was estimated using StringTie, and the FPKM value for gene and transcript levels were calculated using R package Ballgown. Data related to this RNA-seq have been submitted to GEO (approval number = GSE135721 and NCBI tracking system number = 20255902].

**CircRNA *in situ hybridization* and immunostaining.**

Simultaneous mouse *circHomer1* *in situ* hybridization and immunostaining for β3-tubulin, HOMER1B/C, and SMI-312 was carried out using the ViewRNA Cell Plus Assay (ThermoFisher Scientific) per manufacturer’s instructions in 2-3 week differentiated mouse hippocampal neuronal cultures using two probes that can bind to the exon 2/5 splice junction and detection via sequential branched DNA signal amplification. The sequences of mouse *circHomer1a* probes aiming at its exon 5 / exon 2 splice junctions were the following: TTCCACATAG and GGAGCAACCT. The following antibodies were used for co-immunostaining: Homer1b/c - rabbit polyclonal (Synaptic systems, Goettingen, Germany, #160022, 1:500), SMI-312 - mouse monoclonal; (BioLegend, # 837904, 1:1000). The rabbit 488-green used (#SA5-10038, ThermoFisher Scientific, 1:1000) and chicken 647-far red (#SAB4600179, Sigma Aldrich, 1:1000) secondary antibodies were used.

**HuD Motif analysis.**

CircRNA sequences were obtained from Arraystar Inc. The presence of each ARE subtype, and U stretches were analyzed using a previous published script (11). Specifically, the Perl script searches for the following sequences corresponding to HuD motifs 1, 2 and 3 respectively, allowing one mismatch: [CG][CT][CT]TC[CT][CT]TC[TC]C[TC]C, [TG]TTTGTTT[TG][GT]TTT, and TTTTTTTTT[TA]AAA. We also looked for the less restrictive HuD consensus motif [CT]TNN[CT]T[CT].

**RNA Immunoprecipitation (RIP).**

Frontal cortex tissues from adult Wild Type (WT) mice, HUD-OE and HUD KO mice were collected in polysomes lysis buffer (50mM Tris-HCl pH 7.4; 150mM NaCl; 0.5% NP-40; 2mM MgCl2; 1mM EDTA, 1mM EGTA, 10mM NaF, 1mM Na3Vo4; 1mM DTT) supplemented with 200 U/mL RNaseOUT® Ribonuclease Inhibitor (Invitrogen) and EDTA-free protease inhibitor cocktail (Roche, USA) and disaggregated with a Dounce homogenizer. 40uL Dynabeads (Protein G) (Invitrogen) were aliquoted into microfuge tubes and washed 3 times with 20 µL of PBS 1X at RT. 4 µg of the primary antibodies or IgG controls (listed below) where added in a total volume of 200 µL of 1X PBS. Beads were rotated over-night at 4°C. After washing the beads 3 times NT-2 buffer (50mM Tris-HCl, pH 7.4, 150mM NaCl, 0.05% NP-40, 1mM MgCl2) lysates were added to each tube with Ab-bound beads and rotated at 4°C for 4 hours. Beads were washed with 5 min rotation at 4°C, 6 times with NT-2 buffer supplemented with RNaseOUT (100U/mL) and resuspended in 150uL Proteinase K buffer (NT-2 supplemented with 1mg/mL Proteinase K and 1% SDS). Beads were incubated at 55°C, 30 min (vortex occasionally every 5 min); Trizol was added directly to the tubes after Proteinase K treatment for RNA extraction. Aliquots for both RNA and proteins were taken before (input) and after the IP. Primary antibodies: mouse monoclonal anti-myc tag (9B11; Cell Signaling Technology Inc.), while the anti-HuD (E1, sc-28299; Santa Cruz Biotechnology, Inc.) was used for the western blot validation. IgG controls: normal rabbit IgG (sc-2027; Santa Cruz Biotechnology, Inc.); Purified Mouse IgG2a, κ Isotype Ctrl Antibody (#401501; BioLegend). Total RNA was extracted with Trizol® (Invitrogen) according to the manufacturer's recommendations. RNA quality and quantity was determined using the Qubit (Invitrogen) spectrophotometer. WB analysis was performed by SDS–polyacrylamide gel electrophoresis (PAGE). Extracted proteins were boiled in Laemmli sample buffer (0.6 g/100 mL Tris, 2 g/100 mL SDS, 10% glycerol, 1% β-mercaptoethanol, pH 6.8) for 10 min, separated on 10% SDS-PAGE gel and transferred to a PVDF membrane (Bio-Rad) using a liquid transfer apparatus (Bio-Rad). The membranes were treated with a blocking solution containing 5% non-fat dry milk in TBS-T buffer (10 mM Tris-HCl, 100 mM NaCl, 0.1% Tween, pH 7.5) for 1h and incubated overnight with the primary antibodies. Immunoreactivity was detected using the donkey anti-rabbit (GE Healthcare USA, dilution 1:10,000) or anti-mouse (GE Healthcare USA, dilution 1:10,000) secondary peroxidase-conjugated antibodies. The immunoreactive bands were then visualized using the Western Lightning Plus-ECL (PerkinElmer, Inc).

**Statistical Analysis.**

For postmortem measurements, a Univariate General Linear Model, which corrects for RIN, Brain pH, PMI, and Refrigeration Interval, was used (IBM SPSS Statistics 24 – IBM, Armonk, New York). Normalized values were divided to the mean of each control group and the relative to control ratios were plotted as means ± S.E.M. using GraphPad Prism after removing up to 2 outliers using Roots test (Graphpad Software, La Jolla, CA). In all other comparisons between two groups or more than 2 groups where all were compared to the same control group a two-tailed one sample *t*-test was used. In other cases of more than 2 groups were all groups were compared to each other a one-way Analysis of Variance (ANOVA) with correction for multiple comparisons was used. In cases where group variances were significantly different from each another, adjustments were made to the degrees of freedom ( Welch’s correction for t-tests and Huynh-Feldt correction for ANOVAs). For correlations Spearman correlation coefficients and two-tailed p-values were calculated. Chi-squared was used for analysis of differences between expected and observed frequencies. **All circRNA and RNA measurements in postmortem samples and stem cell-derived neuronal cultures (qRT-PCR and microarray) were done blindly and the identity of all samples was only revealed during data analysis.**

**Supplementary Methods References**

1. Weick JP, Groth RD, Isaksen AL, Mermelstein PG. Interactions with PDZ proteins are required for L-type calcium channels to activate cAMP response element-binding protein-dependent gene expression. J Neurosci. 2003;23:3446-56.

2. Brigman JL, Daut RA, Wright T, Gunduz-Cinar O, Graybeal C, Davis MI, et al. GluN2B in corticostriatal circuits governs choice learning and choice shifting. Nat Neurosci. 2013;16:1101-10.

3. Brigman JL, Wright T, Talani G, Prasad-Mulcare S, Jinde S, Seabold GK, et al. Loss of GluN2B-containing NMDA receptors in CA1 hippocampus and cortex impairs long-term depression, reduces dendritic spine density, and disrupts learning. J Neurosci. 2010;30:4590-600.

4. Brigman JL, Mathur P, Harvey-White J, Izquierdo A, Saksida LM, Bussey TJ, et al. Pharmacological or genetic inactivation of the serotonin transporter improves reversal learning in mice. Cereb Cortex. 2010;20:1955-63.

5. Boese AS, Saba R, Campbell K, Majer A, Medina S, Burton L, et al. MicroRNA abundance is altered in synaptoneurosomes during prion disease. Mol Cell Neurosci. 2016;71:13-24.

6. Rao A, Steward O. Evidence that protein constituents of postsynaptic membrane specializations are locally synthesized: analysis of proteins synthesized within synaptosomes. J Neurosci. 1991;11:2881-95.

7. Floruta CM, Du R, Kang H, Stein JL, Weick JP. Default Patterning Produces Pan-cortical Glutamatergic and CGE/LGE-like GABAergic Neurons from Human Pluripotent Stem Cells. Stem Cell Reports. 2017;9:1463-76.

8. Weick JP, Liu Y, Zhang SC. Human embryonic stem cell-derived neurons adopt and regulate the activity of an established neural network. Proc Natl Acad Sci U S A. 2011;108(50):20189-94

9. Zhang SC, Wernig M, Duncan ID, Brustle O, Thomson JA. In vitro differentiation of transplantable neural precursors from human embryonic stem cells. Nat Biotechnol. 2001;19:1129-33.

10. Hoffman GE, Hartley BJ, Flaherty E, Ladran I, Gochman P, Ruderfer DM, et al. Transcriptional signatures of schizophrenia in hiPSC-derived NPCs and neurons are concordant with post-mortem adult brains. Nat Commun. 2017;8:2225.

11. Bolognani F, Contente-Cuomo T, Perrone-Bizzozero NI. Novel recognition motifs and biological functions of the RNA-binding protein HuD revealed by genome-wide identification of its targets. Nucleic Acids Res. 2010;38:117-30.

**SUPPLEMENTARY FIGURE LEGENDS**

**Supplementary Figure 1** Hierarchical clustering analysis and Sanger sequencing for primer validation. Hierarchical clustering analysis of circRNA array data in 100 OFC RNA samples treated with RNase-R for linear RNA digestion. Example of circRNA array raw image is also shown.

**Supplementary Figure 2** Pathway analysis of host genes of altered in SCZ and BD circRNAs. **a**-**b** Schematic showing the molecular pathways formed by the host genes of altered in BD (**a**) and SCZ (**b**) circRNAs based on ingenuity pathway analysis. Information on molecular expression/interactions/relationships are shown in the graph. **c** Sequencing validation of *circHomer1a* splice junction following qRT-PCR. Exon 5 and exon 2 boundaries shown below.

**Supplementary Figure 3** qRT-PCR validation of circRNA and mRNA normalizers and *HOMER1* mRNA expression in the OFC. **a**-**c** Mean ± S.E.M relative to the mean of unaffected Controls *circTulp4/CDR1as* (**a** - calculated as geometric mean of both circRNAs – no additional normalization), *18S rRNA* (**b**), and *HOMER1* mRNA (**c**) expression in BD (blue circles), SCZ (red circles), and Control (green circles) samples from the OFC. *p < 0.05, based on a Univariate General Linear Model corrected for RIN, PMI, RI, and brain pH. **d** Correlation between relative to Control changes in *circHomer1a* and *HOMER1* mRNA expression in the OFC of patients with BD and SCZ as well as unaffected Controls. Spearman correlation coefficient and two-tailed p-values are shown in the graph.

**Supplementary Figure 4** qRT-PCR validation of circRNA and mRNA normalizers and *HOMER1* mRNA expression in the DLPFC. **a**-**c** Mean ± S.E.M relative to the mean of unaffected Controls *circTulp4/CDR1as* (**a** - calculated as geometric mean of both circRNAs – no additional normalization), circADAM22 (**b** – normalized to the geometric mean of *circTulp4* and *CDR1as*) , and *18S rRNA* (**c**) expression in BD (blue circles), SCZ (red circles), and Control (green circles) samples from the DLPFC. **d** Correlation between relative to Control changes in *circHomer1a* and *HOMER1* mRNA expression in the DLPFC of patients with BD and SCZ as well as unaffected Controls. Spearman correlation coefficient and two-tailed p-values are shown in the graph.

**Supplementary Figure 5** Comparison of neuronal gene expression in iPS cell-derived neuronal cultures from patients with BD and SCZ. **a-d** Mean ± SEM relative mRNA expression (based on qRT-PCR and normalized to *18S rRNA*) for *PAX6* (**a**), *DCX* (**b**), *NPTX2* (**c**), and *GAD1* (**d**) in in iPSC-derived BD patient and Control (N=3 Control and 4 BD, Cohort from Mount Sinai School of Medicine) and iPS cell-derived SCZ patient and Control (N=4 Control and 4 SCZ subjects, Cohort from Massachusetts General Hospital) NPs and 6 week differentiated neurons. p > 0.05, *p < 0.05, two-tailed one sample t-test relative to the Control of the same developmental time-point. In all bar graphs the individual replicates are shown within the graph.

**Supplementary Figure 6** Comparison of human and mouse mature *circHomer1a* sequences. Results from NCBI blast alignment of the mature *circHomer1a* sequences for human and mouse (93% sequence conservation).

**Supplementary Figure 7** Cellular and subcellular expression of mouse *circTulp4*. **a** Mean ± SEM relative to the mean expression in D18 neurons) *circTulp4* levels (normalized to *18S rRNA*) in mouse cortical neurons of D18 and D28 of differentiation and mouse cortical astrocytes. **p < 0.01, ***p < 0.001, based on ANOVA with correction for multiple comparison. **b** Mean ± SEM relative *circTulp4* expression (based on qRT-PCR, without normalization) in mouse OFC nuclear, crude synaptosomal (SYN), and cytoplasmic/soluble (S2) subcellular fragments. In all bar graphs the number of replicates is shown within the graph. **c** Mean ± SEM relative to IgG control *Homer1a*, *Homer1b*, and *Homer1c* mRNA isoform levels based on qRT-PCR (normalized to *18S rRNA*) following RIP (anti-Myc) in human HuD-OE-Myc and WT littermates (WT-Ctrl). ^#^0.10 > p > 0.05,*p< 0.05, two-tailed one samples *t*-test. **d-f** Mean ± SEM relative to WT *Homer1a* (**d**), *Homer1b* (**e**), and *Homer1c* (**f**) mRNA isoforms levels (based on qRT-PCR and normalized to *18S rRNA*) in frontal cortex synaptosomes and total frontal cortex isolates (PFC total) from HuD-OE and HUD-KO mice. *p< 0.05, **p < 0.01, two-tailed one samples t-test. In all bar graphs the individual replicates or the number of replicates is shown within the graph.

**Supplementary Figure 8** Map of *circHomer1a* shRNA and control vectors and lentiviral-mediated knockdown of *circHomer1a* expression in mouse neuronal cultures. **a-b** Maps of and scrambled control shRNA (**a**) *circHomer1a* shRNA (**b**) lentiviral vectors used in our study. Notice that while GFP is expressed from a human synapsin (SYN) promoter, shRNAs are driven from a general mouse (U6) promoter. **c-d** Mean ± SEM relative to scrambled shRNA control (*sh-Control*) *circHomer1a* (**c**) and *Homer1a,b,c* mRNA isoform (**d**) levels (based on qRT-PCR and normalized to *18S rRNA*) after shRNA-mediated *circHomer1a* knockdown (*sh-circHomer1*) in mouse neuronal cultures. **p < 0.01, two-tailed one sample t-test relative to *sh-Control* mean, The number of replicates is shown within the graphs.

**Supplementary Figure 9** No changes in cage and open field behavior in following knockdown of *circHomer1a* in mouse OFC. **a** Mean ± SEM number of home cage beam brakes during dark and light periods in mice with OFC injections with either *sh-Control* or *sh-circHomer1*. In all bar graphs the individual replicates or the number of replicates is shown within the graph. **b** Representative traces of the final 10 minutes of open field activity in *sh-Control* (blue) and *sh-circHomer1* (pink) mice. **c-e** Mean ± SEM average velocity (**c**), duration spent in the border versus the center of the open field (**d**), and distance traveled in the border versus center of the open field (**e**) for *sh-Control* (blue) and *sh-circHomer1* (pink) mice. In all bar graphs the individual replicates or the number of replicates is shown within the graph.


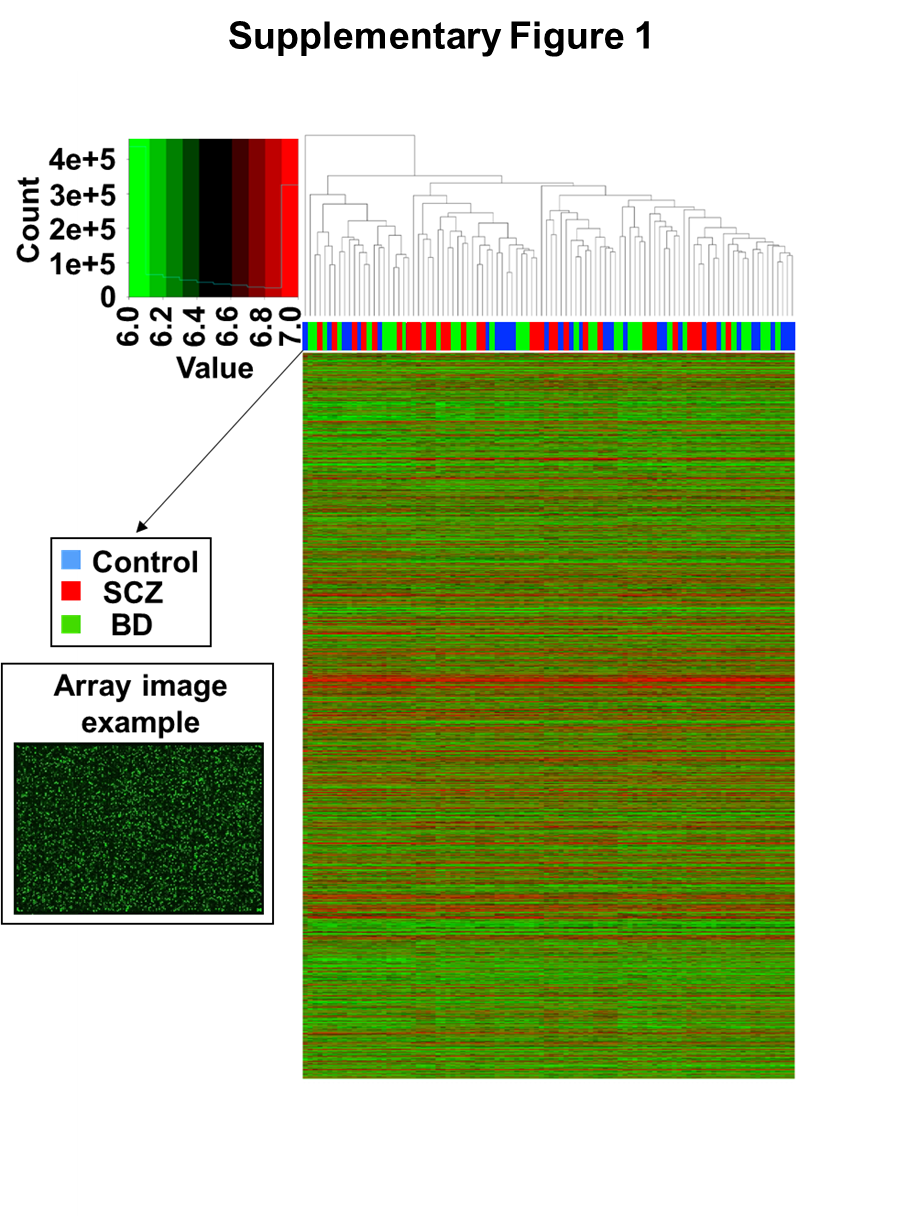


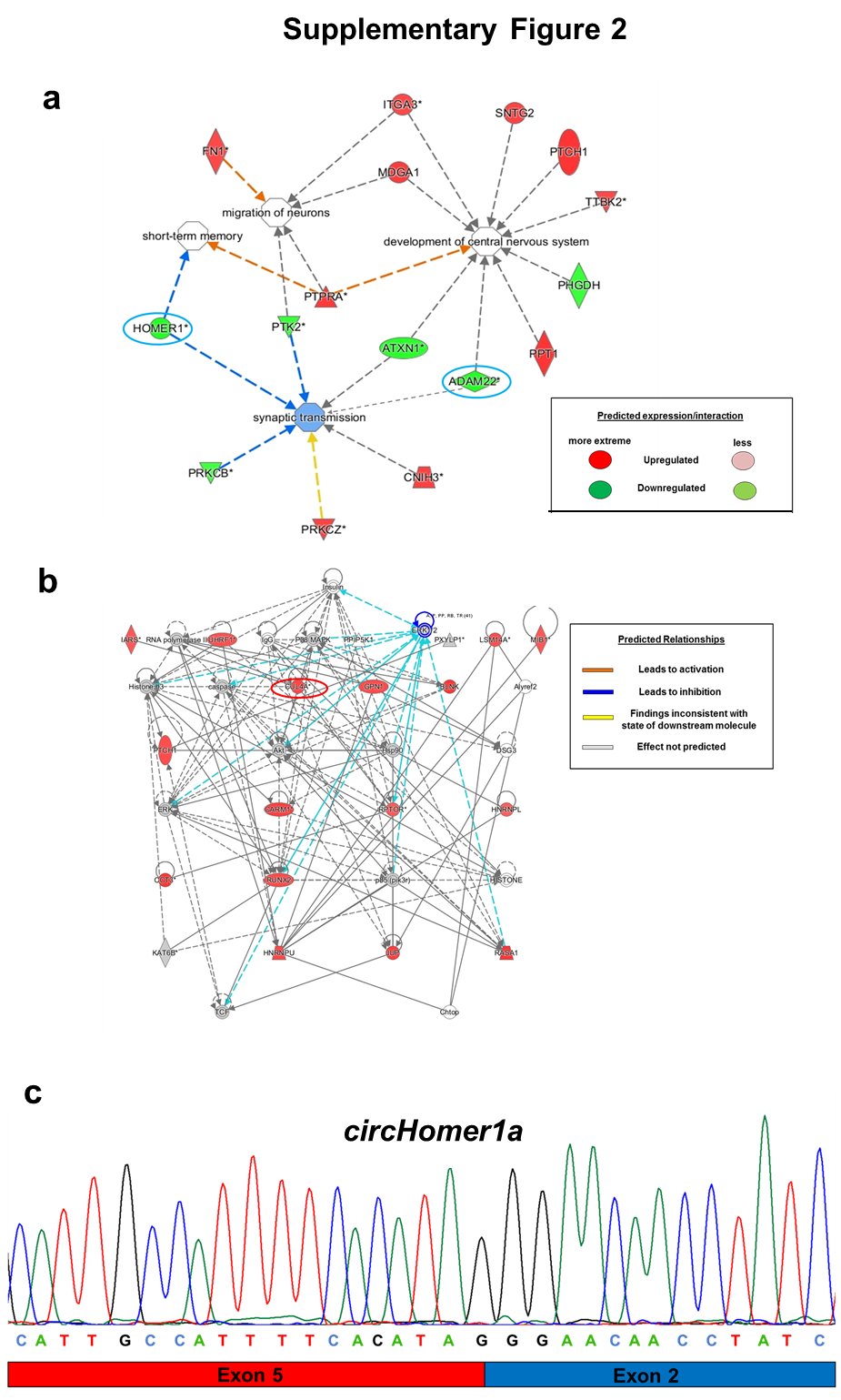


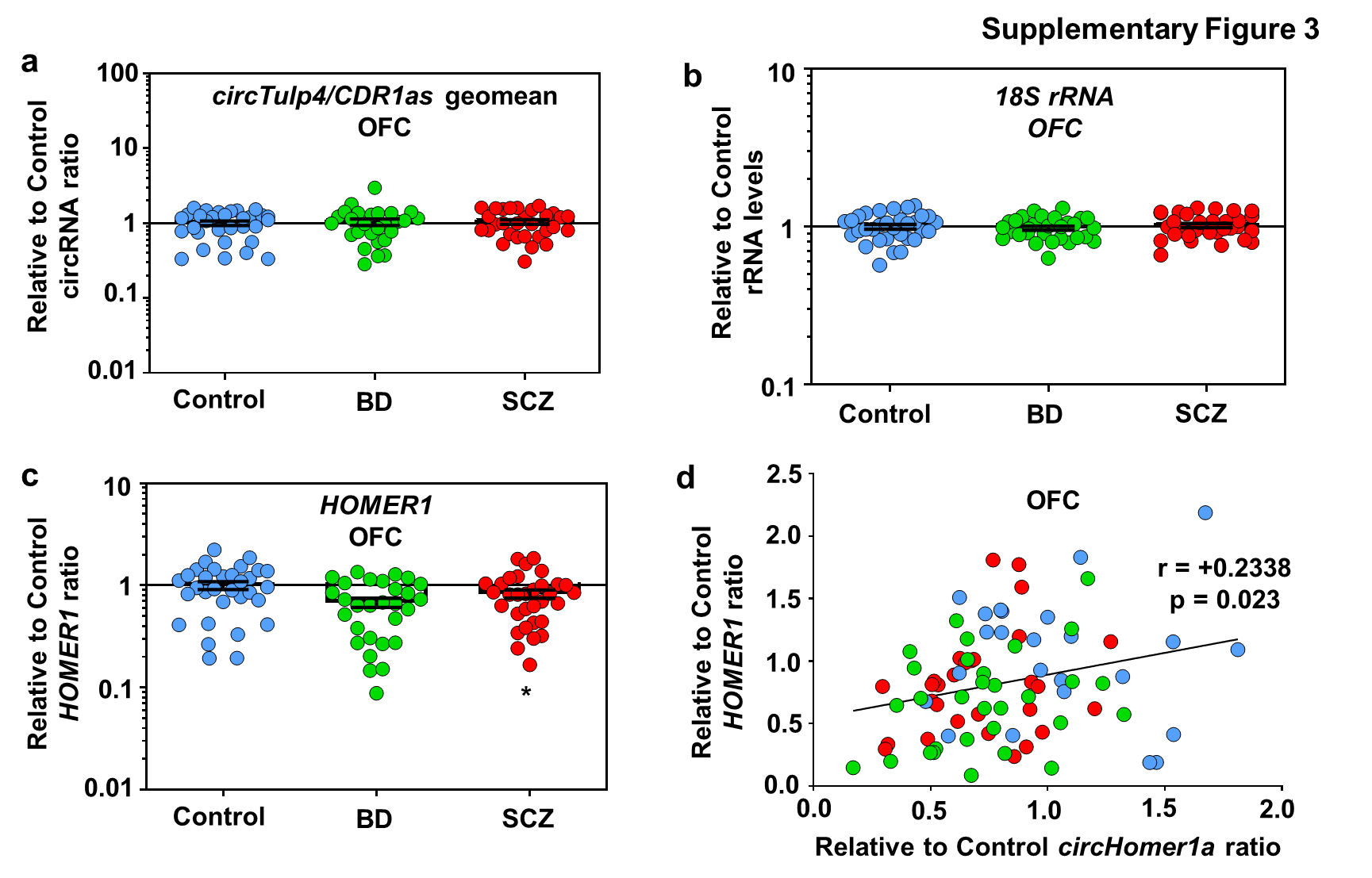


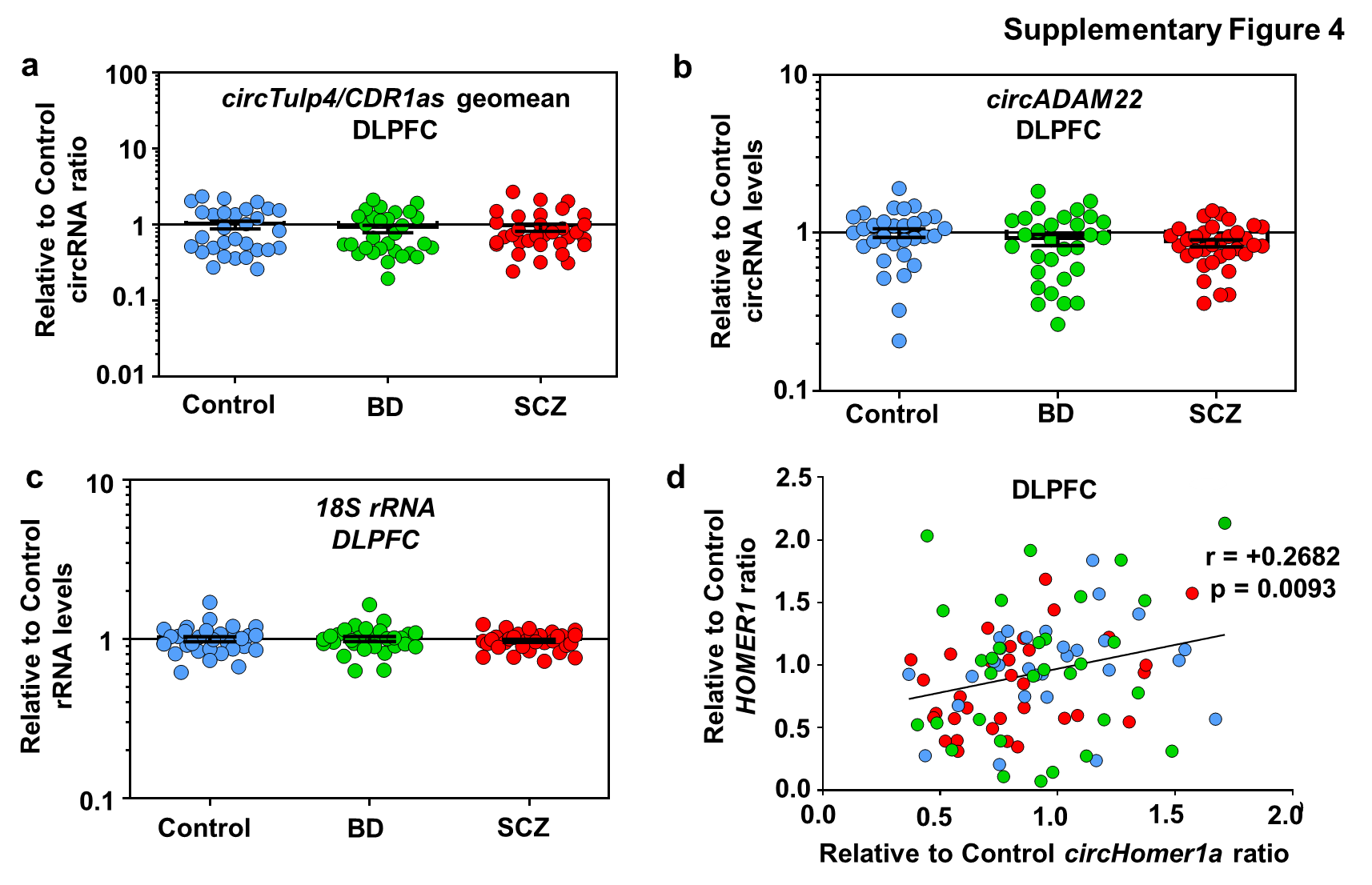


**
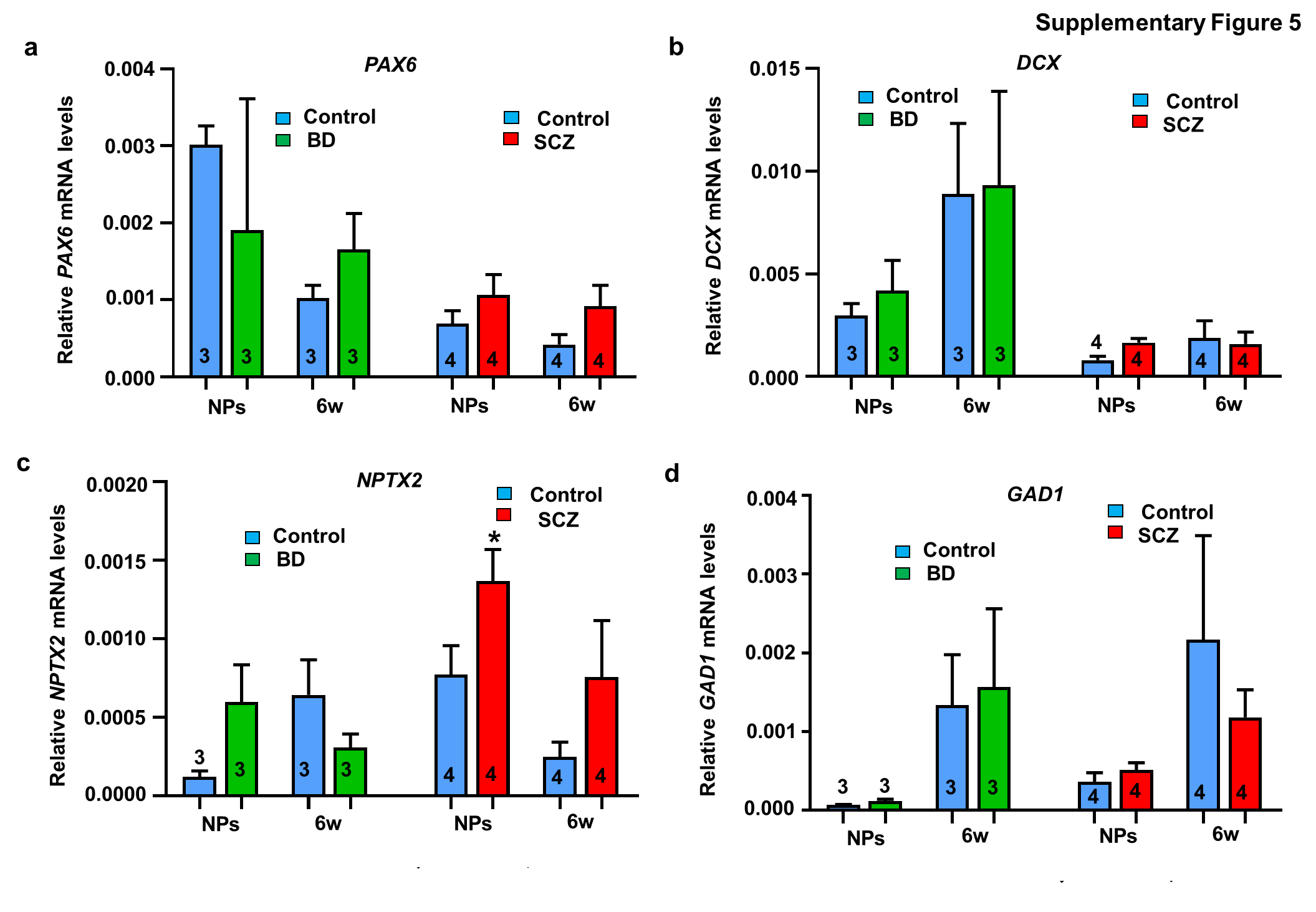
**

**
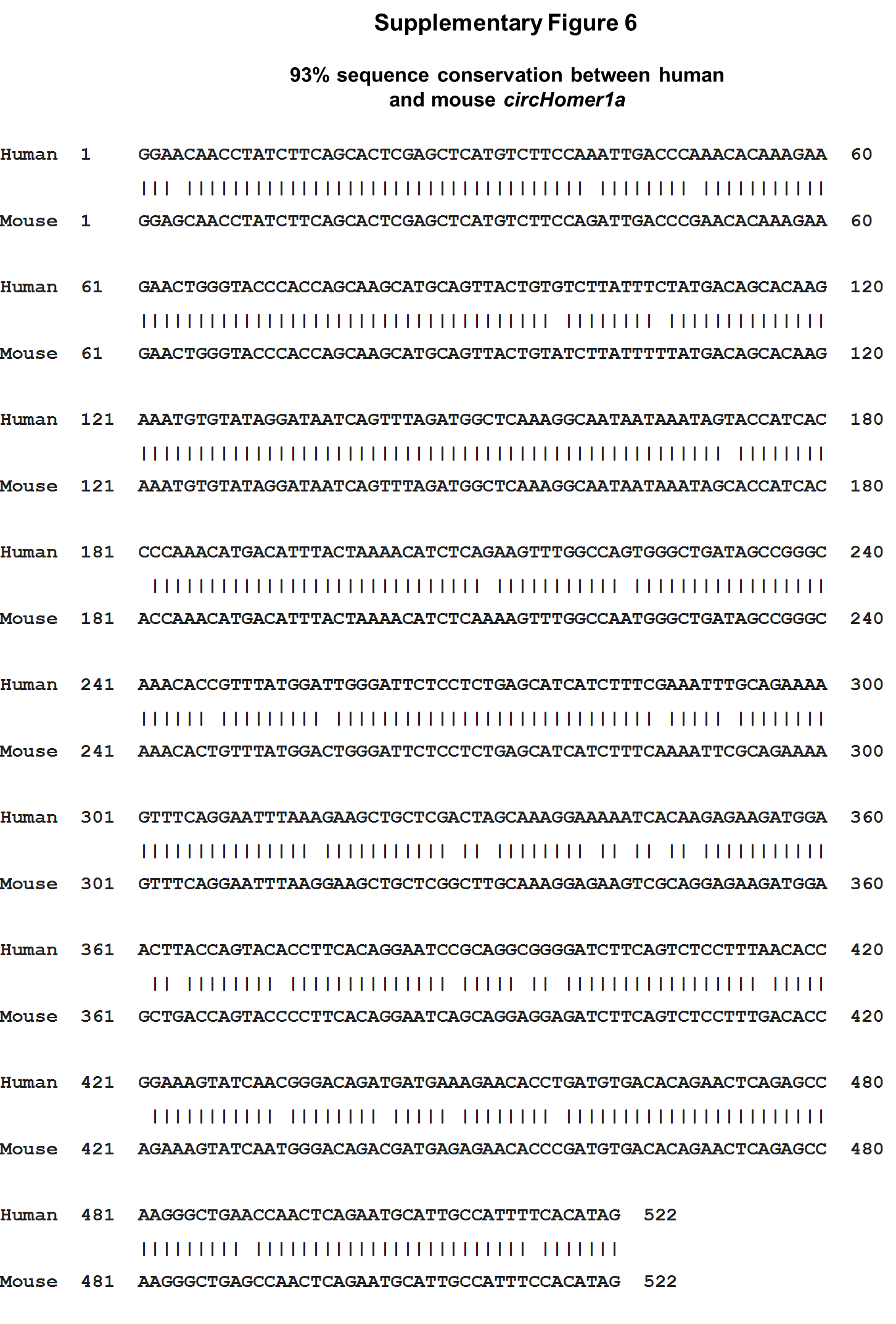
**

**
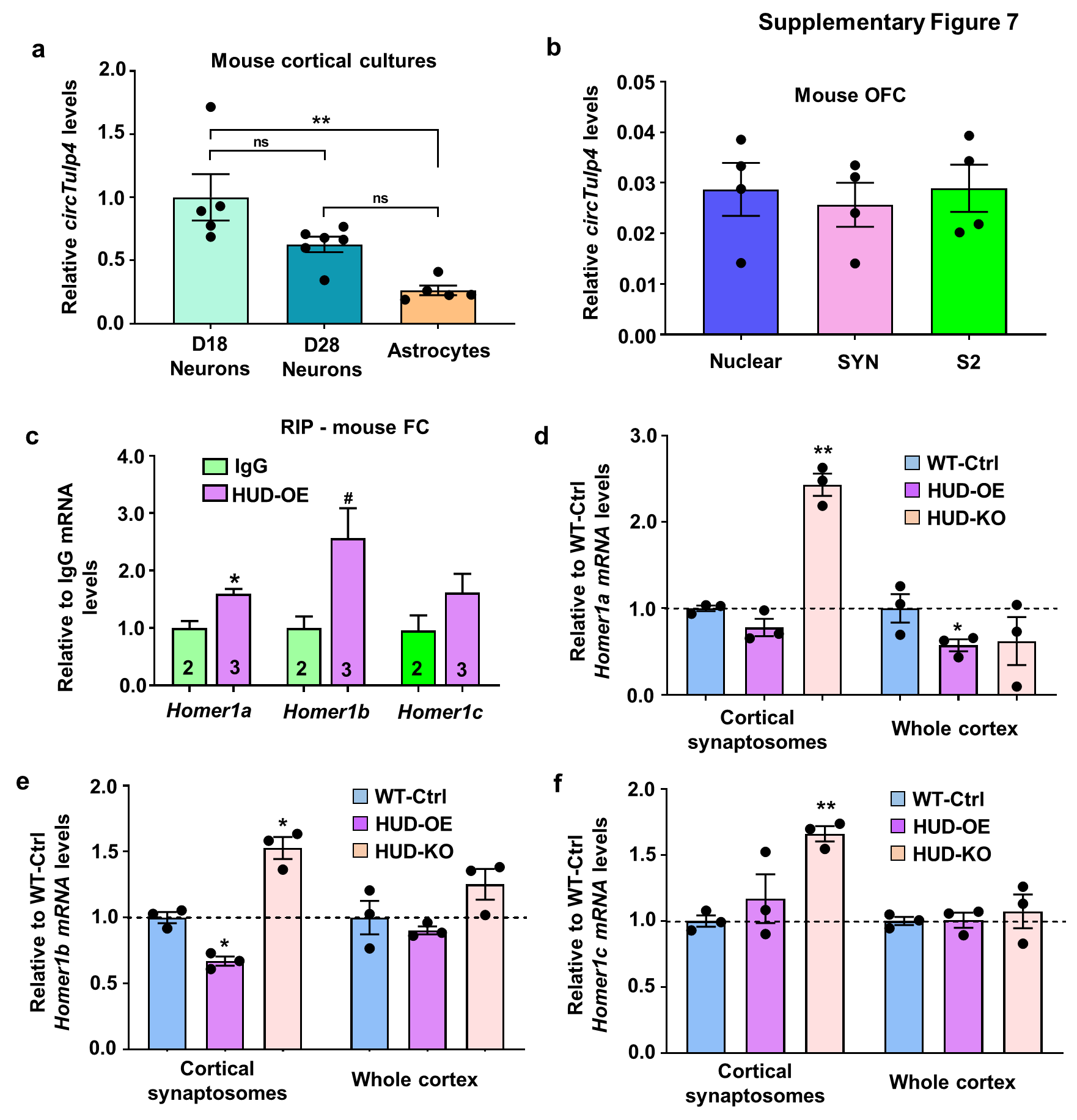
**

**
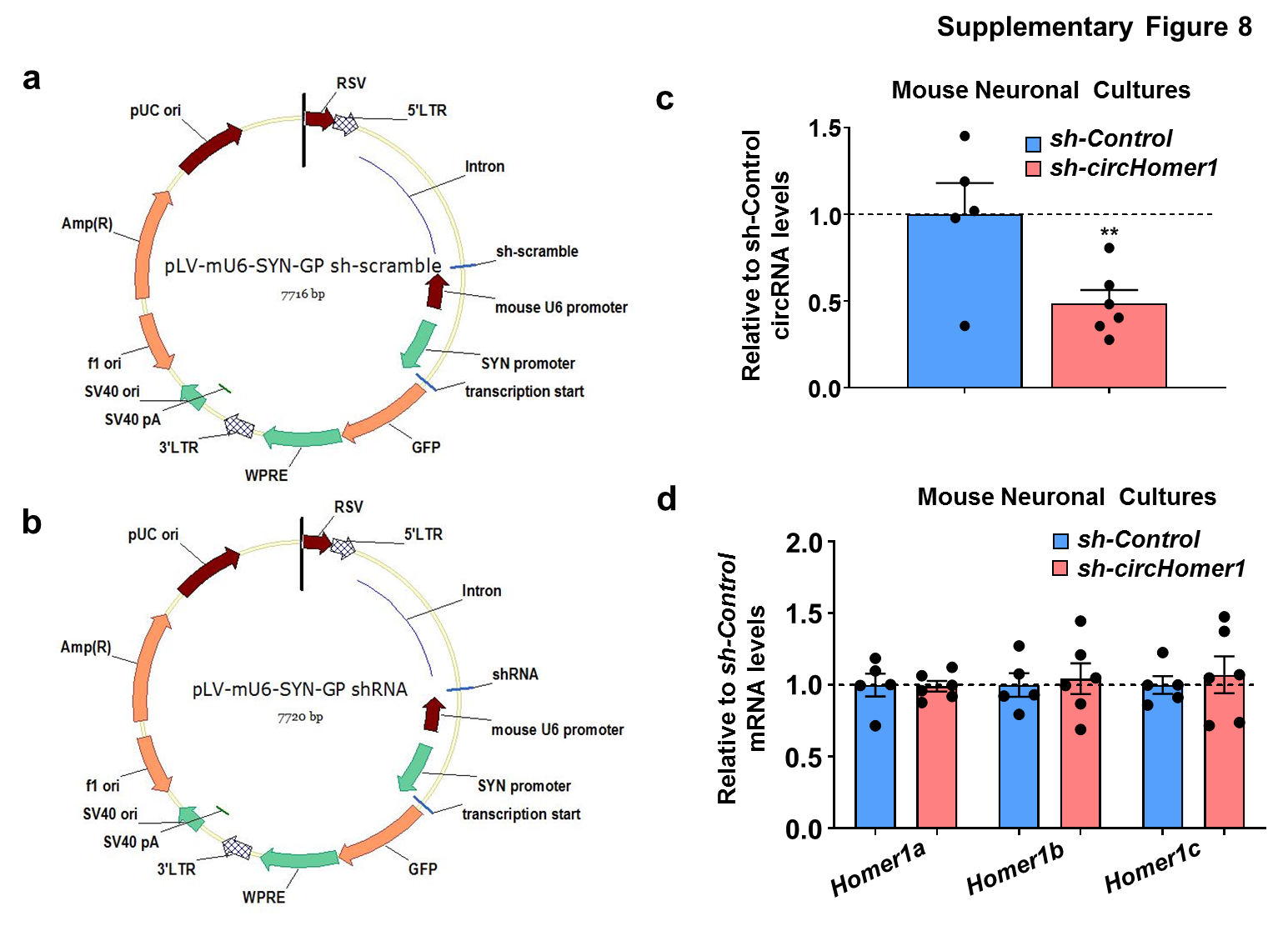
**

**
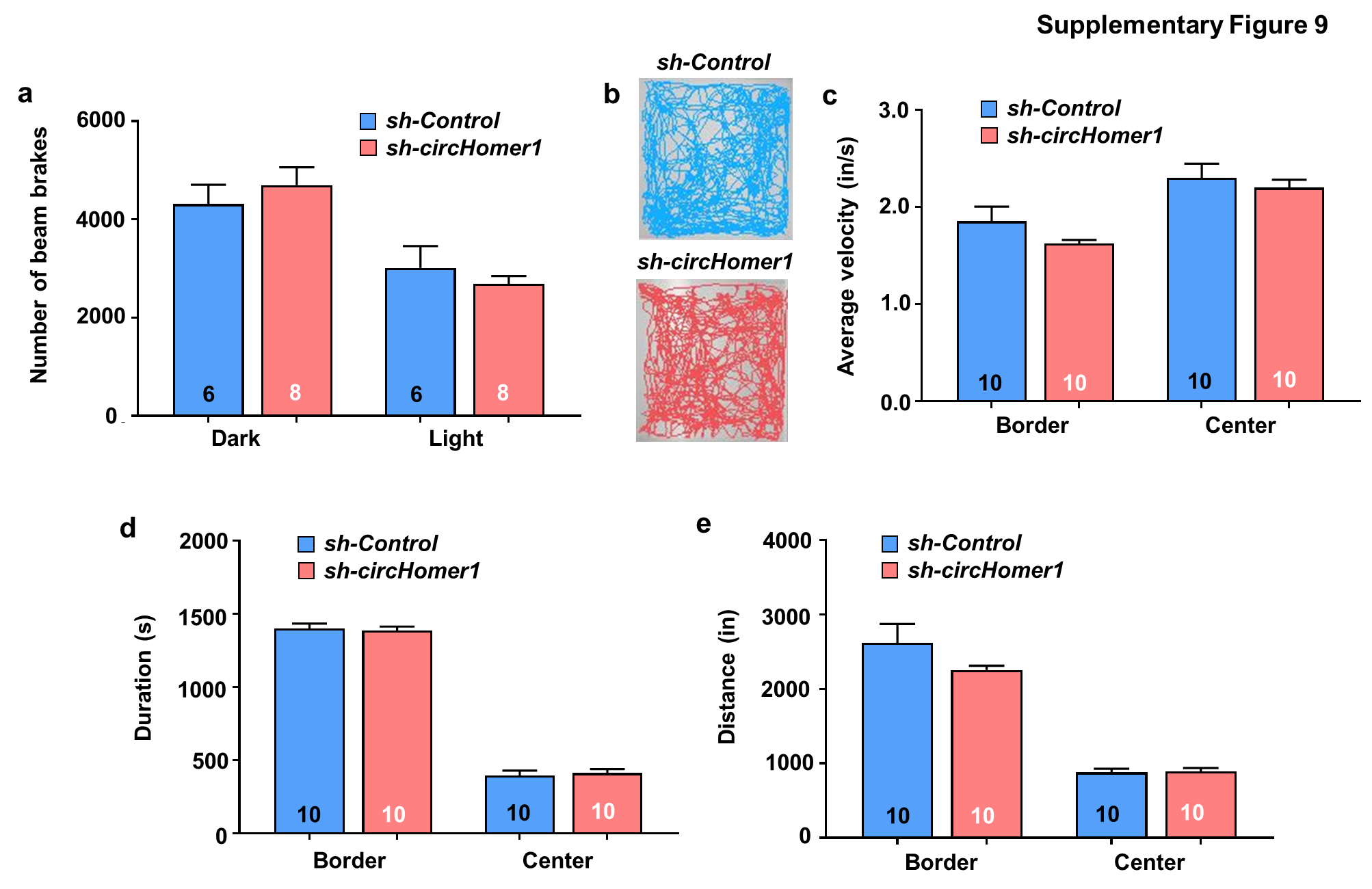
**

**SUPPLEMENTARY TABLE LEGENDS**

**Supplementary Table 1** Summary of demographic information for postmortem OFC cohort. Table showing summarized demographic information for postmortem interval (PMI), RNA integrity number (RIN), brain pH, refrigeration interval (Refr. Int.), age, and sex, in Control, SCZ, and BD subjects. Results are presented as mean ± S.E.M.

**Supplementary Table 2** Significantly altered circRNAs in the OFC of subjects with BD. Table showing the Arraystar circRNA array circRNA ID, the relative to Control fold change and uncorrected p-value in BD, the highest to lowest circRNA expression rank (Rank, smaller numbers represent higher in expression circRNAs), the circRNA alias based on circBase (http://circbase.mdc-berlin.de) or other circRNA databases, the host gene symbol and best transcript ID, the chromosome (Chr) number and strand, and the transcript start and end chromosomal coordinates (tx Start and tx End) for BD-altered circRNAs (more than 1.25 fold change, p < .05, top 5,000 highly expressed circRNAs included only).

**Supplementary Table 3** Significantly altered circRNAs in the OFC of subjects with SCZ. Table showing the Arraystar circRNA array circRNA ID, the relative to Control fold change and uncorrected p-value in SCZ, the highest to lowest circRNA expression rank (Rank, smaller numbers represent higher in expression circRNAs), the circRNA alias based on circBase (http://circbase.mdc-berlin.de) or other circRNA databases, the host gene symbol and best transcript ID, the chromosome (Chr) number and strand, and the transcript start and end chromosomal coordinates (tx Start and tx End) for SCZ-altered circRNAs (more than 1.25 fold change, p < .05, top 5,000 highly expressed circRNAs included only).

**Supplementary Table 4** Information on circRNA and mRNA primers. Table showing the gene name, species, forward and reverse primer sequences and TaqMan assay IDs for human and mouse circRNA and mRNA qRT-PCR measurements.

**Supplementary Table 5** Effects of demographics on OFC *circHomer1a* expression. a, Table showing the Spearman coefficients, 95% confidence intervals, and two-tailed p-values for correlations between relative to Control changes in OFC circHomer1a levels in BD and SCZ (and various postmortem demographics. Only duration of illness appears to be significantly associated with OFC changes in *circHomer1a* expression (highlighted in red).

**Supplementary Table 1**

| Diagnosis  (N) | Control  (N=34) | Schizophrenia (N=34) | Bipolar Disorder (N=32) |
| --- | --- | --- | --- |
| PMI (Mean ± S.E.M) | 29.47 ± 2.20 | 31.94 ± 2.61 | 37.88 ± 3.24 |
| RIN (Mean ± S.E.M) | 7.22 ± 0.15 | 7.36 ± 0.10 | 7.32 ± 0.15 |
| Brain pH (Mean ± S.E.M) | 6.61 ± 0.05 | 6.48 ± 0.04 | 6.46 ± 0.05 |
| RI (Mean ± S.E.M) | 4.15 ± 0.51 | 6.06 ± 0.70 | 10.15 ± 1.81 |
| Age (Mean ± S.E.M) | 43.74 ± 1.26 | 42.47 ± 1.45 | 45.13 ± 1.83 |
| Sex | 25 M / 9 F | 25M / 9F | 16M / 16F |

**Supplementary Table 2**

| **Arraystar ID** | **Fold BD** | **p-value BD** | **Rank** | **circRNA Alias** | **circRNA type** | **GeneSymbol** | **Best_transcript** | **Chrom** | **Strand** | **Tx Start** | **Tx End** |
| --- | --- | --- | --- | --- | --- | --- | --- | --- | --- | --- | --- |
| **hsa_circRNA_101833** | **0.5068** | **0.016611** | **318** | **hsa_circ_0039908** | **exonic** | **DUS2** | **NM_017803** | **chr16** | **+** | **68059317** | **68072052** |
| **hsa_circRNA_080968** | **0.5247** | **0.011017** | **604** | **hsa_circ_0080968** | **exonic** | **ADAM22** | **NM_004194** | **chr7** | **+** | **87765294** | **87797542** |
| **hsa_circRNA_103470** | **0.5266** | **0.017859** | **580** | **hsa_circ_0067301** | **exonic** | **PLXND1** | **NM_015103** | **chr3** | **-** | **129280642** | **129280746** |
| **hsa_circRNA_000585** | **0.5961** | **0.043083** | **72** | **hsa_circ_0000515** | **sense overlapping** | **RPPH1** | **NR_002312** | **chr14** | **-** | **20811305** | **20811534** |
| **hsa_circRNA_103542** | **0.6346** | **0.011791** | **2889** | **hsa_circ_0068464** | **exonic** | **EIF4A2** | **NM_001967** | **chr3** | **+** | **186504915** | **186505373** |
| **hsa_circRNA_103902** | **0.6440** | **0.043068** | **1440** | **hsa_circ_0006916** | **exonic** | **HOMER1** | **NM_004272** | **chr5** | **-** | **78734832** | **78752841** |
| **hsa_circRNA_102700** | **0.6588** | **0.034601** | **2614** | **hsa_circ_0000994** | **exonic** | **SLC8A1** | **NM_021097** | **chr2** | **-** | **40655612** | **40657444** |
| **hsa_circRNA_102950** | **0.6669** | **0.034066** | **649** | **hsa_circ_0058794** | **exonic** | **AGAP1** | **NM_014914** | **chr2** | **+** | **236626200** | **236659132** |
| **hsa_circRNA_005232** | **0.6772** | **0.034010** | **3067** | **hsa_circ_0005232** | **exonic** | **SLC8A1** | **ENST00000403092** | **chr2** | **-** | **40655612** | **40657441** |
| **hsa_circRNA_104964** | **0.6977** | **0.023349** | **626** | **hsa_circ_0006502** | **exonic** | **DPH7** | **NM_138778** | **chr9** | **-** | **140458885** | **140459606** |
| **hsa_circRNA_100900** | **0.7021** | **0.046788** | **1312** | **hsa_circ_0002476** | **exonic** | **INTS4** | **NM_033547** | **chr11** | **-** | **77649696** | **77652297** |
| **hsa_circRNA_100311** | **0.7036** | **0.042251** | **802** | **hsa_circ_0000116** | **exonic** | **MAN1A2** | **NM_006699** | **chr1** | **+** | **117944807** | **117948267** |
| **hsa_circRNA_063280** | **0.7194** | **0.008055** | **1500** | **hsa_circ_0063280** | **exonic** | **PICK1** | **NM_012407** | **chr22** | **+** | **38463710** | **38466898** |
| **hsa_circRNA_103730** | **0.7201** | **0.035573** | **1376** | **hsa_circ_0005654** | **exonic** | **PRDM5** | **NM_018699** | **chr4** | **-** | **121675707** | **121732604** |
| **hsa_circRNA_028671** | **0.7231** | **0.012457** | **1207** | **hsa_circ_0028671** | **exonic** | **TAOK3** | **NM_016281** | **chr12** | **-** | **118682698** | **118693460** |
| **hsa_circRNA_104066** | **0.7232** | **0.045509** | **1813** | **hsa_circ_0004032** | **exonic** | **ATXN1** | **NM_000332** | **chr6** | **-** | **16658006** | **16753578** |
| **hsa_circRNA_039187** | **0.7250** | **0.016001** | **870** | **hsa_circ_0039187** | **exonic** | **HERC2P4** | **NR_109773** | **chr16** | **-** | **32190706** | **32190881** |
| **hsa_circRNA_031900** | **0.7255** | **0.029598** | **1240** | **hsa_circ_0031900** | **exonic** | **TMX1** | **NM_030755** | **chr14** | **+** | **51712030** | **51716483** |
| **hsa_circRNA_000686** | **0.7298** | **0.011736** | **739** | **hsa_circ_0000686** | **intronic** | **QPRT** | **ENST00000449759** | **chr16** | **+** | **29677910** | **29678100** |
| **hsa_circRNA_001846** | **0.7332** | **0.031858** | **163** | **hsa_circ_0000520** | **sense overlapping** | **RPPH1** | **NR_002312** | **chr14** | **-** | **20811436** | **20811559** |
| **hsa_circRNA_103252** | **0.7334** | **0.006861** | **2399** | **hsa_circ_0008472** | **exonic** | **ATXN10** | **NM_013236** | **chr22** | **+** | **46096161** | **46125470** |
| **hsa_circRNA_005414** | **0.7347** | **0.017850** | **1120** | **hsa_circ_0005414** | **sense overlapping** | **CMTM7** | **NM_138410** | **chr3** | **+** | **32479446** | **32483505** |
| **hsa_circRNA_012123** | **0.7392** | **0.002665** | **2081** | **hsa_circ_0012123** | **exonic** | **ATP6V0B** | **uc001clf.3** | **chr1** | **+** | **44441761** | **44443967** |
| **hsa_circRNA_002919** | **0.7422** | **0.014389** | **1193** | **hsa_circ_0002919** | **exonic** | **AP3S1** | **NM_001284** | **chr5** | **+** | **115202366** | **115205825** |
| **hsa_circRNA_002635** | **0.7498** | **0.021458** | **2520** | **hsa_circ_0002635** | **exonic** | **B3GALTL** | **NM_194318** | **chr13** | **+** | **31789187** | **31803431** |
| **hsa_circRNA_004738** | **0.7582** | **0.021254** | **894** | **hsa_circ_0004738** | **exonic** | **RANBP17** | **NM_022897** | **chr5** | **+** | **170667931** | **170669824** |
| **hsa_circRNA_023691** | **0.7614** | **0.018773** | **1153** | **hsa_circ_0023691** | **exonic** | **CLNS1A** | **NM_001293** | **chr11** | **-** | **77327195** | **77348851** |
| **hsa_circRNA_020094** | **0.7679** | **0.032223** | **1382** | **hsa_circ_0020094** | **exonic** | **ATRNL1** | **NM_207303** | **chr10** | **+** | **116879948** | **116925405** |
| **hsa_circRNA_030788** | **0.7692** | **0.031915** | **2483** | **hsa_circ_0030788** | **exonic** | **NALCN** | **NM_052867** | **chr13** | **-** | **101997616** | **102031004** |
| **hsa_circRNA_102053** | **0.7708** | **0.029296** | **980** | **hsa_circ_0043284** | **exonic** | **TADA2A** | **NM_001488** | **chr17** | **+** | **35802664** | **35804870** |
| **hsa_circRNA_100024** | **0.7767** | **0.017415** | **1359** | **hsa_circ_0009135** | **exonic** | **NPHP4** | **NM_015102** | **chr1** | **-** | **6021853** | **6029319** |
| **hsa_circRNA_072697** | **0.7769** | **0.007801** | **1174** | **hsa_circ_0072697** | **exonic** | **PPWD1** | **NM_015342** | **chr5** | **+** | **64863339** | **64868113** |
| **hsa_circRNA_100445** | **0.7795** | **0.035666** | **662** | **hsa_circ_0016404** | **exonic** | **TATDN3** | **NM_001042552** | **chr1** | **+** | **212977661** | **212977993** |
| **hsa_circRNA_104700** | **0.7833** | **0.049133** | **2996** | **hsa_circ_0005273** | **exonic** | **PTK2** | **NM_005607** | **chr8** | **-** | **141710989** | **141716304** |
| **hsa_circRNA_103306** | **0.7852** | **0.036979** | **3474** | **hsa_circ_0064555** | **exonic** | **SATB1** | **NM_002971** | **chr3** | **-** | **18419661** | **18462483** |
| **hsa_circRNA_000121** | **0.7862** | **0.042309** | **151** | **hsa_circ_0000121** | **antisense** | **PHGDH** | **NM_006623** | **chr1** | **-** | **120285606** | **120286575** |
| **hsa_circRNA_406403** | **0.7865** | **0.022463** | **4718** | **n/a** | **exonic** | **KLHL24** | **NM_017644** | **chr3** | **+** | **183388821** | **183390272** |
| **hsa_circRNA_101004** | **0.7920** | **0.003713** | **2432** | **hsa_circ_0000375** | **exonic** | **IFFO1** | **NM_080730** | **chr12** | **-** | **6657590** | **6657991** |
| **hsa_circRNA_402150** | **1.2516** | **0.023152** | **4430** | **n/a** | **exonic** | **SNTG2** | **NM_018968** | **chr2** | **+** | **1093881** | **1168869** |
| **hsa_circRNA_009366** | **1.2527** | **0.049267** | **765** | **hsa_circ_0009366** | **exonic** | **PRKCZ** | **NM_002744** | **chr1** | **+** | **2066700** | **2087531** |
| **hsa_circRNA_020845** | **1.2549** | **0.034348** | **1131** | **hsa_circ_0020845** | **exonic** | **TSSC2** | **NR_024248** | **chr11** | **+** | **3424822** | **3426193** |
| **hsa_circRNA_000938** | **1.2564** | **0.021925** | **3094** | **hsa_circ_0000938** | **sense overlapping** | **ZNF223** | **NM_013361** | **chr19** | **+** | **44564901** | **44605080** |
| **hsa_circRNA_004797** | **1.2567** | **0.041325** | **135** | **hsa_circ_0004797** | **exonic** | **CNIH3** | **NM_152495** | **chr1** | **+** | **224868659** | **224922408** |
| **hsa_circRNA_103120** | **1.2612** | **0.025186** | **1934** | **hsa_circ_0002113** | **exonic** | **IFNGR2** | **NM_005534** | **chr21** | **+** | **34793786** | **34805178** |
| **hsa_circRNA_407331** | **1.2619** | **0.038061** | **1330** | **n/a** | **intronic** | **ENOX2** | **ENST00000370935** | **chrX** | **-** | **129917520** | **129943235** |
| **hsa_circRNA_104391** | **1.2633** | **0.021792** | **2010** | **hsa_circ_0080451** | **exonic** | **POM121** | **NM_172020** | **chr7** | **+** | **72398922** | **72400649** |
| **hsa_circRNA_067327** | **1.2673** | **0.043071** | **1198** | **hsa_circ_0067327** | **exonic** | **ALG1L2** | **NM_001136152** | **chr3** | **+** | **129811943** | **129813356** |
| **hsa_circRNA_104503** | **1.2675** | **0.029931** | **1763** | **hsa_circ_0082582** | **exonic** | **TRIM24** | **NM_003852** | **chr7** | **+** | **138203933** | **138255748** |
| **hsa_circRNA_102983** | **1.2683** | **0.042995** | **738** | **hsa_circ_0005265** | **exonic** | **PTPRA** | **NM_002836** | **chr20** | **+** | **2928627** | **2945848** |
| **hsa_circRNA_100685** | **1.2705** | **0.048160** | **505** | **hsa_circ_0020080** | **exonic** | **FAM160B1** | **NM_020940** | **chr10** | **+** | **116590610** | **116608496** |
| **hsa_circRNA_001126** | **1.2765** | **0.027174** | **844** | **hsa_circ_0001126** | **exonic** | **PTPRA** | **NM_002836** | **chr20** | **+** | **2903852** | **2945848** |
| **hsa_circRNA_020846** | **1.2822** | **0.024476** | **888** | **hsa_circ_0020846** | **exonic** | **TSSC2** | **NR_024248** | **chr11** | **+** | **3424822** | **3427945** |
| **hsa_circRNA_102492** | **1.2893** | **0.012964** | **3786** | **hsa_circ_0050205** | **exonic** | **MEF2BNB-MEF2B** | **NM_005919** | **chr19** | **-** | **19260034** | **19261573** |
| **hsa_circRNA_406768** | **1.2906** | **0.017424** | **3716** | **n/a** | **exonic** | **MDGA1** | **NM_153487** | **chr6** | **-** | **37617884** | **37620116** |
| **hsa_circRNA_091722** | **1.2944** | **0.022759** | **2724** | **hsa_circ_0091722** | **exonic** | **MAGEA3** | **NM_005362** | **chrX** | **-** | **151934651** | **151936377** |
| **hsa_circRNA_105026** | **1.3030** | **0.016941** | **4248** | **hsa_circ_0007733** | **exonic** | **DOCK11** | **NM_144658** | **chrX** | **+** | **117788564** | **117788924** |
| **hsa_circRNA_400994** | **1.3047** | **0.038476** | **1733** | **n/a** | **exonic** | **ZDHHC17** | **uc001syj.2** | **chr12** | **+** | **77214837** | **77216311** |
| **hsa_circRNA_011286** | **1.3077** | **0.043759** | **953** | **hsa_circ_0011286** | **exonic** | **SERINC2** | **NM_018565** | **chr1** | **+** | **31899500** | **31907527** |
| **hsa_circRNA_102984** | **1.3094** | **0.038191** | **585** | **hsa_circ_0006117** | **exonic** | **PTPRA** | **NM_002836** | **chr20** | **+** | **2944917** | **2945848** |
| **hsa_circRNA_401857** | **1.3144** | **0.049299** | **2004** | **n/a** | **exonic** | **INTS2** | **NM_020748** | **chr17** | **-** | **59981820** | **59996874** |
| **hsa_circRNA_104878** | **1.3212** | **0.022844** | **2485** | **hsa_circ_0003458** | **exonic** | **PTBP3** | **NM_005156** | **chr9** | **-** | **115013208** | **115060196** |
| **hsa_circRNA_103505** | **1.3235** | **0.013883** | **564** | **hsa_circ_0067911** | **exonic** | **PHC3** | **NM_024947** | **chr3** | **-** | **169863210** | **169863309** |
| **hsa_circRNA_103507** | **1.3251** | **0.005555** | **623** | **hsa_circ_0067913** | **exonic** | **PHC3** | **NM_024947** | **chr3** | **-** | **169863210** | **169890500** |
| **hsa_circRNA_036088** | **1.3525** | **0.039553** | **1556** | **hsa_circ_0036088** | **exonic** | **RPLP1** | **NM_001003** | **chr15** | **+** | **69745158** | **69747884** |
| **hsa_circRNA_407202** | **1.3600** | **0.026164** | **602** | **n/a** | **exonic** | **PTCH1** | **NM_000264** | **chr9** | **-** | **98231032** | **98232213** |
| **hsa_circRNA_062142** | **1.3667** | **0.040979** | **489** | **hsa_circ_0062142** | **exonic** | **TPTEP1** | **NR_001591** | **chr22** | **+** | **17117929** | **17128675** |
| **hsa_circRNA_011883** | **1.3679** | **0.013004** | **3229** | **hsa_circ_0011883** | **exonic** | **PPT1** | **NM_000310** | **chr1** | **-** | **40544231** | **40546159** |
| **hsa_circRNA_011977** | **1.3831** | **0.019309** | **719** | **hsa_circ_0011977** | **exonic** | **PPIH** | **NM_006347** | **chr1** | **+** | **43142254** | **43142429** |
| **hsa_circRNA_016459** | **1.3911** | **0.019396** | **2561** | **hsa_circ_0016459** | **exonic** | **KCNK2** | **NM_014217** | **chr1** | **+** | **215259710** | **215298093** |
| **hsa_circRNA_104511** | **1.3919** | **0.047126** | **1499** | **hsa_circ_0082708** | **exonic** | **SLC37A3** | **NM_032295** | **chr7** | **-** | **140055467** | **140069482** |
| **hsa_circRNA_402116** | **1.4055** | **0.027466** | **766** | **n/a** | **exonic** | **HIF3A** | **NM_022462** | **chr19** | **+** | **46832463** | **46834530** |
| **hsa_circRNA_003785** | **1.4346** | **0.031918** | **548** | **hsa_circ_0003785** | **sense overlapping** | **XLOC_012658** | **TCONS_00026319** | **chr18** | **+** | **32902351** | **32907027** |

**Supplementary Table 3**

| **Arraystar ID** | **Fold SCZ** | **p-value SCZ** | **Rank** | **circRNA Alias** | **circRNA type** | **GeneSymbol** | **Best_transcript** | **Chrom** | **Strand** | **Tx Start** | **Tx End** |
| --- | --- | --- | --- | --- | --- | --- | --- | --- | --- | --- | --- |
| **hsa_circRNA_104964** | **0.7502** | **0.023410** | **626** | **hsa_circ_0006502** | **exonic** | **DPH7** | **NM_138778** | **chr9** | **-** | **140458885** | **140459606** |
| **hsa_circRNA_000686** | **0.7558** | **0.008499** | **739** | **hsa_circ_0000686** | **intronic** | **QPRT** | **ENST00000449759** | **chr16** | **+** | **29677910** | **29678100** |
| **hsa_circRNA_104833** | **0.7820** | **0.021129** | **1133** | **hsa_circ_0002191** | **exonic** | **C9orf3** | **NM_032823** | **chr9** | **+** | **97535283** | **97563284** |
| **hsa_circRNA_400082** | **0.7970** | **0.048014** | **652** | **hsa_circ_0092371** | **intronic** | **PLEKHG4B** | **ENST00000283426** | **chr5** | **+** | **173662** | **173882** |
| **hsa_circRNA_404495** | **1.2660** | **0.016965** | **4751** | **n/a** | **intronic** | **KLF17** | **uc009vxf.1** | **chr1** | **+** | **44568737** | **44571186** |
| **hsa_circRNA_405174** | **1.2790** | **0.029899** | **3147** | **n/a** | **intronic** | **FARP1** | **ENST00000319562** | **chr13** | **+** | **98923104** | **98923600** |
| **hsa_circRNA_405781** | **1.2820** | **0.034410** | **3525** | **n/a** | **intronic** | **HNRNPL** | **ENST00000221419** | **chr19** | **-** | **39328342** | **39329036** |
| **hsa_circRNA_002554** | **1.2871** | **0.034490** | **4483** | **hsa_circ_0002554** | **exonic** | **ACTR8** | **NM_022899** | **chr3** | **-** | **53908237** | **53910107** |
| **hsa_circRNA_004561** | **1.3013** | **0.032679** | **4146** | **hsa_circ_0004561** | **intronic** | **TRIQK** | **ENST00000517540** | **chr8** | **-** | **93929156** | **93941802** |
| **hsa_circRNA_087497** | **1.3144** | **0.045888** | **2923** | **hsa_circ_0087497** | **exonic** | **IARS** | **NM_002161** | **chr9** | **-** | **95027210** | **95027835** |
| **hsa_circRNA_102217** | **1.3148** | **0.034760** | **1370** | **hsa_circ_0000813** | **exonic** | **RPTOR** | **NM_020761** | **chr17** | **+** | **78865519** | **78867665** |
| **hsa_circRNA_401929** | **1.3183** | **0.035669** | **1997** | **n/a** | **exonic** | **MIB1** | **NM_020774** | **chr18** | **+** | **19371334** | **19379935** |
| **hsa_circRNA_000148** | **1.3800** | **0.027218** | **1898** | **hsa_circ_0000148** | **sense overlapping** | **UCK2** | **NM_012474** | **chr1** | **+** | **165878068** | **165878169** |
| **hsa_circRNA_405718** | **1.3895** | **0.020911** | **2919** | **n/a** | **exonic** | **UHRF1** | **NM_013282** | **chr19** | **+** | **4947116** | **4951008** |
| **hsa_circRNA_407202** | **1.3997** | **0.020495** | **602** | **n/a** | **exonic** | **PTCH1** | **NM_000264** | **chr9** | **-** | **98231032** | **98232213** |
| **hsa_circRNA_001536** | **1.4034** | **0.022284** | **2837** | **hsa_circ_0001536** | **intronic** | **FAM13B** | **ENST00000502471** | **chr5** | **-** | **137371579** | **137372196** |
| **hsa_circRNA_402188** | **1.4136** | **0.026991** | **904** | **n/a** | **exonic** | **GPN1** | **NM_007266** | **chr2** | **+** | **27862909** | **27870810** |
| **hsa_circRNA_102509** | **1.4161** | **0.043682** | **2001** | **hsa_circ_0006446** | **exonic** | **LSM14A** | **NM_015578** | **chr19** | **+** | **34685382** | **34687668** |
| **hsa_circRNA_005526** | **1.4252** | **0.023616** | **3330** | **hsa_circ_0005526** | **intronic** | **RUNX2** | **ENST00000478660** | **chr6** | **+** | **45459677** | **45460699** |
| **hsa_circRNA_092377** | **1.4447** | **0.016002** | **3043** | **hsa_circ_0000200** | **sense overlapping** | **HNRNPU** | **NM_004501** | **chr1** | **-** | **245018859** | **245019265** |
| **hsa_circRNA_016346** | **1.4589** | **0.043070** | **1513** | **hsa_circ_0016346** | **exonic** | **KCNH1** | **NM_002238** | **chr1** | **-** | **210970849** | **211192598** |
| **hsa_circRNA_001241** | **1.4626** | **0.040250** | **1210** | **hsa_circ_0000508** | **intronic** | **CUL4A** | **ENST00000326335** | **chr13** | **+** | **113869912** | **113870043** |
| **hsa_circRNA_407148** | **1.4655** | **0.037625** | **1778** | **n/a** | **intergenic** | **n/a** | **n/a** | **chr9** | **-** | **5859467** | **5867902** |
| **hsa_circRNA_100656** | **1.4823** | **0.027901** | **1838** | **hsa_circ_0004896** | **exonic** | **EXOSC1** | **NM_016046** | **chr10** | **-** | **99196947** | **99197507** |
| **hsa_circRNA_011538** | **1.4846** | **0.012143** | **1099** | **hsa_circ_0011538** | **exonic** | **ZMYM4** | **NM_005095** | **chr1** | **+** | **35824525** | **35855699** |
| **hsa_circRNA_405685** | **1.4947** | **0.017745** | **1644** | **n/a** | **sense overlapping** | **RP11-49K24.4** | **ENST00000592747** | **chr18** | **-** | **44617544** | **44623775** |
| **hsa_circRNA_003907** | **1.5093** | **0.035796** | **1547** | **hsa_circ_0003907** | **intronic** | **FARP1** | **ENST00000319562** | **chr13** | **+** | **98923108** | **98923600** |
| **hsa_circRNA_102445** | **1.5181** | **0.026606** | **2113** | **hsa_circ_0004552** | **exonic** | **CARM1** | **NM_199141** | **chr19** | **+** | **11015626** | **11019883** |
| **hsa_circRNA_043602** | **1.5316** | **0.040689** | **1138** | **hsa_circ_0043602** | **exonic** | **JUP** | **uc010wfs.2** | **chr17** | **-** | **39679868** | **39925459** |
| **hsa_circRNA_001587** | **1.5461** | **0.024474** | **364** | **hsa_circ_0000979** | **intronic** | **XLOC_001374** | **TCONS_00003590** | **chr2** | **+** | **19042277** | **19042456** |
| **hsa_circRNA_407205** | **1.5973** | **0.030421** | **661** | **n/a** | **sense overlapping** | **RP11-535M15.1** | **ENST00000423924** | **chr9** | **+** | **99481790** | **99483412** |
| **hsa_circRNA_404595** | **1.6075** | **0.035314** | **2445** | **n/a** | **intronic** | **CCT3** | **ENST00000295688** | **chr1** | **-** | **156303463** | **156304503** |
| **hsa_circRNA_007507** | **1.7728** | **0.018331** | **843** | **hsa_circ_0007507** | **exonic** | **RASA1** | **NM_002890** | **chr5** | **+** | **86627164** | **86633908** |
| **hsa_circRNA_066869** | **1.8702** | **0.042714** | **979** | **hsa_circ_0066869** | **sense overlapping** | **TMEM39A** | **NM_018266** | **chr3** | **-** | **119176864** | **119182471** |
| **hsa_circRNA_001350** | **2.1277** | **0.038105** | **588** | **hsa_circ_0000253** | **intronic** | **BLNK** | **NR_047681** | **chr10** | **-** | **97999787** | **97999925** |

**Supplementary Table 4**

| **Gene name** | **Species** | **Primer sequence or TaqMan ID** |
| --- | --- | --- |
| *h_circHomer1a-F* | Human | TCAACGGGACAGATGATGAA |
| *h_circHomer1a-R* | Human | TTGTGTTTGGGTCAATTTGG |
| *h_circCUL4A-F* | Human | CTATGGAGGTCACGTCCCG |
| *h_circCUL4A-R* | Human | AGAGCCACATGGATGAAACCT |
| *h_CDR1as-F* | Human | ACGTCTCCAGTGTGCTGA |
| *h_CDR1as-R* | Human | CTTGACACAGGTGCCATC |
| *h_circTulp4-F* | Human | GGAGTGGTTGGGGTGACTTT |
| *h_circTulp4-R* | Human | TCAACTGCCATACGAAGCGT |
| *h_circADAM22-F* | Human | TGTTGCTGGCACCAATATCATAA |
| *h_circADAM22-R* | Human | AGTGTCTCCCATTATGCACCC |
| *h_HOMER1* | Human | Hs01029333_m1 |
| *h_18S rRNA* | Human | Hs99999901_s1 |
| *m_circHomer1a-F* | Mouse | TTCCACATAGGGAGCAACC |
| *m_circHomer1a-R* | Mouse | TCTTCTTTGTGTTCGGGTCA |
| *m_cirTulp4-F* | Mouse | TCACTGTCGCAGAGATAGGAGT |
| *m_cirTulp4-R* | Mouse | GGCACTTGATATGTTTGTTTTCC |
| *m_circCREBBP-F* | Mouse | GAATGCCCTACCCTGCTCC |
| *m_circCREBBP-R* | Mouse | TGGGGATCAGCTCATCAGGA |
| *m_Homer1a-F* | Mouse | GAAGTCGCAGGAGAAGATG |
| *m_HOMER1a-R* | Mouse | TGATTGCTGAATTGAATGTGTACC |
| *m_Homer1b-F* | Mouse | ACACCCGATGTGACACAGAA |
| *m_Homer1b-R* | Mouse | CACTGCTTCACATTGGCAGT |
| *m_Homer1c-F* | Mouse | GAGAAGTCGCAGGAGAAGATG |
| *m_Homer1c-R* | Mouse | TTGCTGAACTAGCATGAGAGAG |
| *m_Psma4-207-F* | Mouse | CATCCGGGTTGGTTCTTCT |
| *m_Psma4-207-R* | Mouse | ATTCAAGGACCACGGGAAGT |
| *m_Psma4-201-F* | Mouse | GCTCTGGCTGTTAAGGTGCT |
| *m_Psma4-201-R* | Mouse | CCACTTCCTTTTGCTTGAGG |
| *m_18S rRNA-F* | Mouse | AAGAGGGCTGGAGAACTCAC |
| *m_18S rRNA-R* | Mouse | GCAGCTTGTTGTCTAGACCG |

**Supplementary Table 5**

| **Demographics** | **Spearman r** | **95% confidence**  **interval** | **P (two-tailed)** | **Significant?**  **(alpha = 0.05)** | **Number of XY Pairs** |
| --- | --- | --- | --- | --- | --- |
| **RIN** | 0.1408 | -0.1347 to 0.396 | 0.3008 | No | 56 |
| **PMI** | -0.0245 | -0.2928 to 0.2474 | 0.8578 | No | 56 |
| **BrainPH** | 0.1899 | -0.08479 to 0.4377 | 0.1611 | No | 56 |
| **Refrigerator Interval** | -0.0219 | -0.2905 to 0.2499 | 0.8727 | No | 56 |
| **Age** | -0.1525 | -0.4061 to 0.1228 | 0.2618 | No | 56 |
| **Sex** | -0.0633 | -0.328 to 0.2106 | 0.6428 | No | 56 |
| **Race** | 0.1367 | -0.1387 to 0.3925 | 0.3151 | No | 56 |
| **Lifetime Antipsychotics** | -0.2026 | -0.4484 to 0.07158 | 0.1342 | No | 56 |
| **SuicideStatus** | 0.1891 | -0.0856 to 0.437 | 0.1628 | No | 56 |
| **Hemisphere** | -0.1016 | -0.362 to 0.1734 | 0.4560 | No | 56 |
| **Brain Weight** | 0.0238 | -0.2481 to 0.2922 | 0.8616 | No | 56 |
| **AgeOfOnset** | 0.1055 | -0.1697 to 0.3653 | 0.4392 | No | 56 |
| **Duration of Illness** | -0.2778 | -0.5098 to -0.008159 | 0.0381 | Yes | 56 |
| **Lifetime Alcohol Use** | 0.2149 | -0.06145 to 0.4606 | 0.1151 | No | 55 |
| **Lifetime Drug Use** | 0.1971 | -0.0827 to 0.448 | 0.1532 | No | 54 |
| **Smoking At TOD** | 0.0108 | -0.3145 to 0.3338 | 0.9479 | No | 39 |
| **Time In Hospital** | -0.2401 | -0.4813 to 0.03496 | 0.0775 | No | 55 |
| **Psychotic Feature** | -0.1224 | -0.3847 to 0.1583 | 0.3781 | No | 54 |
